# The Evolution of Epigean and Stygobitic Species of *Koonunga* Sayce, 1907 (Syncarida: Anaspidacea) in Southern Australia, with the Description of Three New Species

**DOI:** 10.1371/journal.pone.0134673

**Published:** 2015-08-26

**Authors:** Remko Leijs, Tessa Bradford, James G. Mitchell, William F. Humphreys, Steven J. B. Cooper, Peter Goonan, Rachael A. King

**Affiliations:** 1 School of Biological Sciences, Flinders University of South Australia, Bedford Park, Adelaide, Australia; 2 South Australian Museum, North Terrace, Adelaide, Australia; 3 School of Biological Sciences and Australian Centre for Evolutionary Biology and Biodiversity, The University of Adelaide, Adelaide, Australia; 4 Western Australian Museum, Welshpool, Australia and School of Animal Biology, University of Western Australia, Perth, Australia; 5 South Australian Environment Protection Authority, Adelaide, Australia; Queensland Museum, AUSTRALIA

## Abstract

Three new species of *Koonunga* were discovered in surface and subterranean waters in southern Australia, and were defined using mtDNA analyses and morphology. The new species are: *Koonunga hornei* Leijs & King; *K*. *tatiaraensis* Leijs & King and *K*. *allambiensis* Leijs & King. Molecular clock analyses indicate that the divergence times of the species are older than the landscape that they currently inhabit. Different scenarios explaining this apparent discrepancy are discussed in the context of the palaeography of the area. A freshwater epigean origin for *Koonunga* is considered the most likely hypothesis, whereby some lineages made the transition to the subterranean environment within the last few million years influenced by significant climatic cooling/drying. We discuss the possibility that one stygobitic lineage secondarily regained some of its body pigmentation as adaptation to increased photic conditions after cave collapse and forming of cenotes during the last glacial maximum.

## Introduction

**Fig 1:**
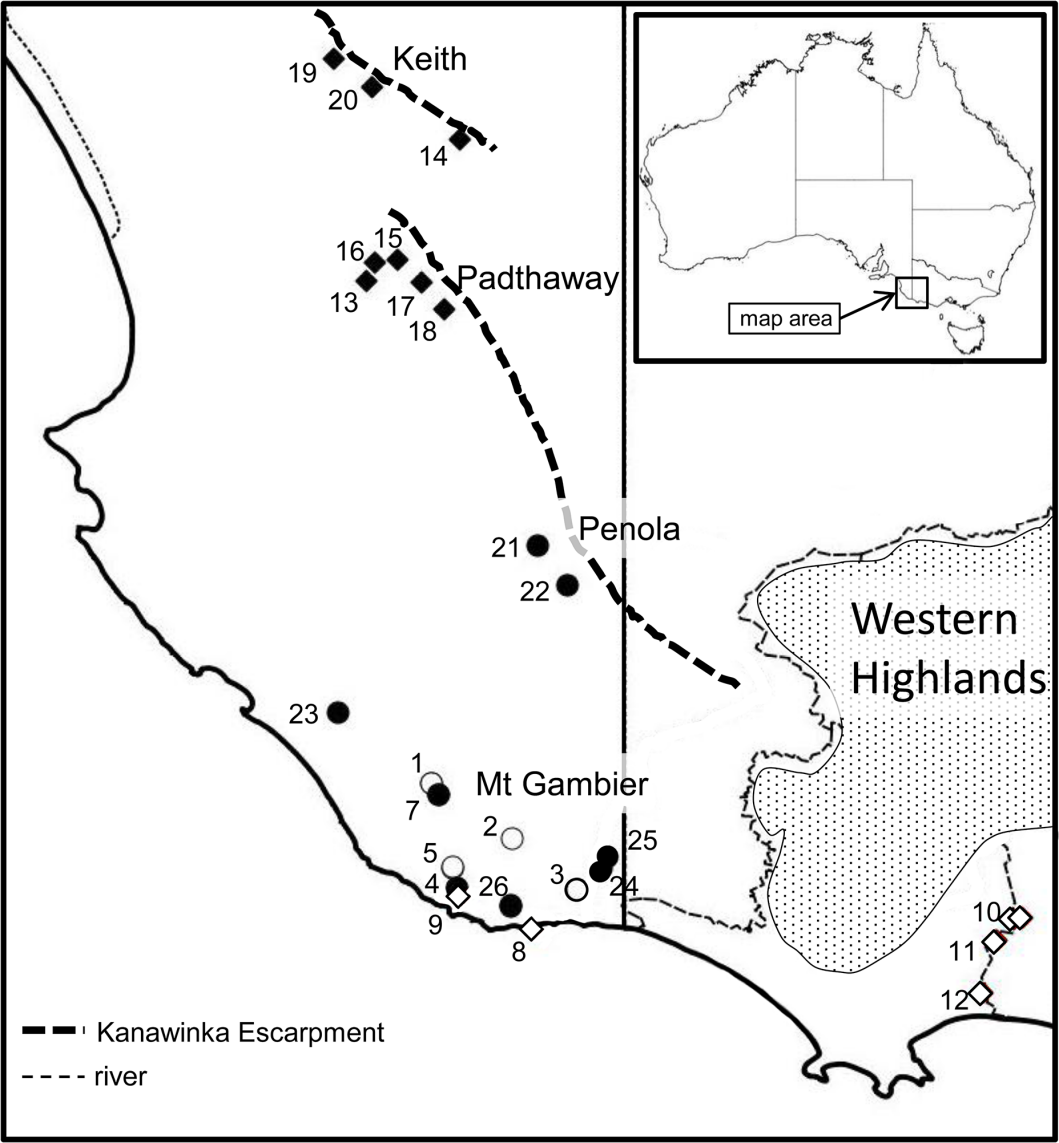
Map of the distribution of the *Koonunga* species treated in this paper. Locality numbers refer to data in Table 1. Open circle: *K*. *crenarum*; closed circles *K*. *hornei* sp. nov.; closed diamonds *K*. *tatiaraensis* sp. nov.; open diamonds *K*. *allambiensis* sp. nov..

The order Anaspidacea Calman, 1904 [1] (Crustacea, Malacostraca, Syncarida) is an ancient taxon, as evidenced by numerous Permian fossils from northern and southern hemisphere marine habitats [2, 3] as well as a single Australian Triassic fossil from the Hawkesbury Sandstone (*Anaspidites antiquus* Chilton 1929) [4]. The latter has a remarkably similar morphology to extant *Anaspides* Thomson, 1894 [5] species [6]. Presently, three of the four families within the Anaspidacea (Anaspididae Thomson, 1983 [7], Koonungidae Sayce, 1907 [8] and Psammaspididae Schminke, 1974 [9]) are endemic to south-east Australia (New South Wales, Victoria, Tasmania and South Australia) and are found in fresh surface water and subterranean habitats [10], while species belonging to the fourth family Stygocarididae Noodt, 1963 [11] occur in South America, as well as in Australia, and are found exclusively in subterranean waters [12]. Here we focus on the species within the family Koonungidae that currently contains two genera: *Micraspides* Nicholls, l93l [13] and *Koonunga* Sayce, 1907 [8]. *Micraspides* is monotypic and its species inhabits freshwater crayfish burrows in western Tasmania, while *Koonunga* currently comprises two species. *Koonunga cursor* Sayce, 1907 [8], is reported to occur in southern Victoria and north-western Tasmania [14, 15] in small permanent to semi-permanent wetlands and pools in irregularly flowing creeks. *Koonunga crenarum* Zeidler, 1985 [16] was described from cenotes (sinkholes) and caves in the Mount Gambier area of South Australia. Several additional undescribed species of *Koonunga* have been reported to occur in Victoria [14], King Island and north-west Tasmania [17]. During a three year project (2008–2011) that aimed to survey South Australian subsurface groundwater dependent ecosystems, new species of *Koonunga* were found in the south-east of South Australia (Fig 1).The habitat in which the new species of *Koonunga* were collected consists of a large semi-continuous unconfined aquifer in the Tertiary Gambier Limestone with a low salinity, total dissolved solids were < 1500 mg/L [18].

The palaeogeographic history of south-east South Australia helps to understand the evolution and the current distribution of the different *Koonunga* species. The Mount Gambier coastal plain, defined by the current coastline and the Kanawinka Escarpment (Fig 1) in the north-east is less than 1.1 My old [19] and was formed by a general and gradual uplift of the area which in turn caused a marine regression. Periodic cycles (100ka) of sea level changes, caused by fluctuations of global ice volume and temperature change [20, 21] deposited a number of parallel calcareous sand dune formations (Bridgewater formation) of decreasing age towards the coast. These sand dune formations roughly indicate past historical coastline positions. The underlying geology of relevance in terms of understanding the distribution of the *Koonunga* species mainly consists of a thick layer of Gambier Limestone, up to 400 m thick in places, which was formed in the Murray and Otway Basins in the Late Miocene-Late Pliocene, > 15 Mya [22].

The flat topography and porous nature of the limestone from Mount Gambier towards the coast means there is no natural surface drainage network, apart from permanent coastal springs and their short drainages [23]. The only surface water in the area is found in cenotes (large collapsed caves), from which the majority of the *K*. *crenarum* specimens were collected. In the northern part of the area surface water is restricted to a number of lagoons with fluctuating water levels due to variable groundwater inflows and rainfall inputs. Especially in the southern part of the area karstification of the Gambier limestone resulted in the forming of caves and cenotes. This karstification may have been accelerated by volcanogenic CO_2_ emissions associated with the Late Pleistocene Mount Gambier eruption around 28ka [23]. Cenotes, caused by ceiling collapses of larger cavities, probably occurred during the Last Glacial Maximum (20 ka) when the sea-level was circa 120 m below the current level [24], which would have greatly reduced groundwater levels [23]. Strontium isotope analyses of stromatolites in the cenotes suggest they flooded with the sea level rise circa 8000 years ago [23].

In this paper we use molecular and morphological data to delineate and describe three new species of *Koonunga*. Species divergence time estimates are obtained from molecular data, and together with historical data on geology and geography, we discuss scenarios for the evolution of these *Koonunga* species.

## Materials and Methods

### Specimen collection and taxa examined

*Koonunga* specimens were collected from the south-east of South Australia in an area that encompassed Keith in the north to Port MacDonnell in the south (Fig 1), as part of a three-year project to study the biodiversity of groundwater in South Australia. In addition, groundwater sampling in the Keith-Bordertown area was done in collaboration with the South Australian Environment Protection Authority. Furthermore, specimens were also collected during a Bush Blitz Survey (Australian Biological Resources Study, Department of Environment, Canberra) in the Lake Condah area of Victoria. Observation bores were sampled with the permission of the Department of Water, Land and Biodiversity Conservation, Adelaide. Sinkholes, caves and wells were sampled with the permission of the landowners. No specific ethics approval was needed for this invertebrate group. The field studies did not involve endangered or protected species. *Koonunga* specimens were collected from surface waters such as creeks, spring fed wetlands and cenotes using a 1 mm mesh sieve and from groundwater using variable diameter weighted plankton nets (mesh size 0.1 mm) that fit groundwater observation wells. These weighted plankton nets were used to filter the water column in the wells repeatedly, dislodging material and fauna from the bottom and walls. Mop traps were used to attract specimens. These traps consisted of a few strands of cotton fibre from a mop that provided a surface for biofilms that subsequently attracted species of higher trophic levels in the ecosystem. These traps were left *in situ* for days to several months. Additionally the Bou-Rouch [25] method of pumping and filtering 50–100 litres of water from 0.5–1 m below the surface was used for collecting from gravel in springs and cave debris. Samples were sorted within a few hours and specimens were preserved in absolute ethanol.

The holotype (SAMA C13286) and additional material of *Koonunga crenarum* present in the collections of the South Australian Museum (SAMA C3990-C3992) were examined for comparative purposes. Sayce (1908) did not designate holotype material of *K*. *cursor*, which he collected from Mullum Creek in Ringwood, near Melbourne. However, syntype specimens collected by Sayce from this locality (NMV J1046), were loaned and examined from the collections at Museum Victoria.

Water quality parameters of groundwater wells were recorded using a Hach Hydrolab MS5 water quality sonde. Parameters measured were: Temperature (°C), Specific Conductance (mS/cm), Salinity (ppt), pH (units) and Dissolved Oxygen saturation DO (% saturation).

### Molecular methods

Genomic DNA was extracted from ethanol preserved specimens and frozen specimens of the Australian Biological Tissue Collection, South Australian Museum. DNA was extracted from pereopods (thoracopods), or from the entire body of small specimens using DNAzol (Molecular Research Center, Cincinnati, Ohio) [26] with the following modifications. Before extraction, ethanol preserved material was completely desiccated, and prior to centrifugation the homogenate was incubated at room temperature for two hours with proteinase K (400 μg/mL; Sigma, St. Louis, MO) after which DNA was precipitated overnight at -20°C with 100% ethanol.

A 658 bp region of the mitochondrial Cytochrome oxidase subunit 1 (COI) gene was PCR-amplified using a combination of universal primers: M414 (forward, 5’-GGT CAA CAA ATC ATA AAG ATA TTG G-3’, alias LCO1490, [27]), M423 (reverse, 5’-TAA ACT TCA GGG TGA CCA AAA AAT CA-3’, alias LCO2198, [27]). PCR amplifications were carried out in 25- μL volumes with approximately 100 ng genomic DNA, 4 mM MgCl_2_, 0.20 mM dNTPs, 1 × PCR buffer (Applied Biosystems), 6 pmol of each primer (Geneworks) and 0.5 U of Ampli *Taq* Gold (Applied Biosystems). PCR amplification was performed under the following conditions: 94° C for 9 min, then 34 cycles of 94°C for 45 s; annealing 48°C for 45 s; 72°C for 60 s; with a final elongation step at 72°C for 6 min. Sequence data were obtained by sending 50μL PCR products to Macrogen, Korea, for purification and Sanger sequencing on both strands using the same primers that were used for PCR amplification. ChromasPro version 1.34 (Technelysium Pty Ltd.) was used to edit chromatogram files, to determine a consensus sequence from both strands, and to align sequences. MEGA [28] was used for analyses of amino acids.

### Molecular analyses

Sequence data were obtained for up to five specimens per locality. Phylogenetic analyses of aligned sequence data were carried out using the program PAUP* version 4.0b8 [29], MrBayes ver. 3.2.4 [30] and BEAST version 1.7.2 [31]. *Anaspides tasmaniae* (GenBank accession DQ310660) was used as an outgroup in the analyses. PAUP* was used for neighbour joining analyses of uncorrected sequence divergence. BEAST was used to enable estimates of divergence times to be derived using relaxed molecular clock methods. As fossils are not known for *Koonunga*, a mean rate of 0.0105 substitutions per site per million years [32] was used as a prior in analyses with an uncorrelated log-linear relaxed molecular clock. This rate replaces the often used invertebrate clock rate of Brower [33] and should be considered as a better estimate, because it is based on more independent data and improved models of sequence evolution. We are aware of the limitations of using a ‘borrowed’ clock rate, but because it is the average of a number of independent rate calibrations, for the moment, we consider it the best option to use for our divergence estimates. We used a Yule process of speciation for analyses of species relationships and a coalescence model with constant population size for separate analyses of relationships within the individual species. The analyses were performed applying unlinked data partitions for each of the codons for the COI gene and using a general time-reversible model [34], with a proportion of invariant sites and unequal rates among sites [35], modelled with a gamma distribution (GTR + I + G). Tracer v1.5[36] was used to check that the effective sample size (ESS) of the parameters after the MrBayes and BEAST runs were larger than 100 and to check for convergence among multiple runs. Bayesian analyses were run for 5 million generations, with two independent runs. Results were combined after removing the first 25 percent (relative burnin) of the generated data.

### Morphological methods

For the taxonomic descriptions, alcohol preserved specimens were softened in glycerol for 1–2 days before dissection of appendages along the left side of the body. Illustrations were produced using a drawing tube attachment to a Nikon Eclipse 80i microscope. Type material has been lodged with the South Australian Museum (SAMA) and Museum Victoria (NMV) (Lake Condah species).

For the classification of the setae we used Garm [37].

### Nomenclatural acts

The electronic edition of this article conforms to the requirements of the amended International Code of Zoological Nomenclature, and hence the new names contained herein are available under that Code from the electronic edition of this article. This published work and the nomenclatural acts it contains have been registered in ZooBank, the online registration system for the ICZN. The ZooBank LSIDs (Life Science Identifiers) can be resolved and the associated information viewed through any standard web browser by appending the LSID to the prefix "http://zoobank.org/". The LSID for this publication is: urn:lsid:zoobank.org:pub: CC0523AC-3D0B-412A-95E3-6CBCE6122A73. The electronic edition of this work was published in a journal with an ISSN, and has been archived and is available from the following digital repositories: PubMed Central, LOCKSS.

## Results

### Molecular phylogeny

**Fig 2:**
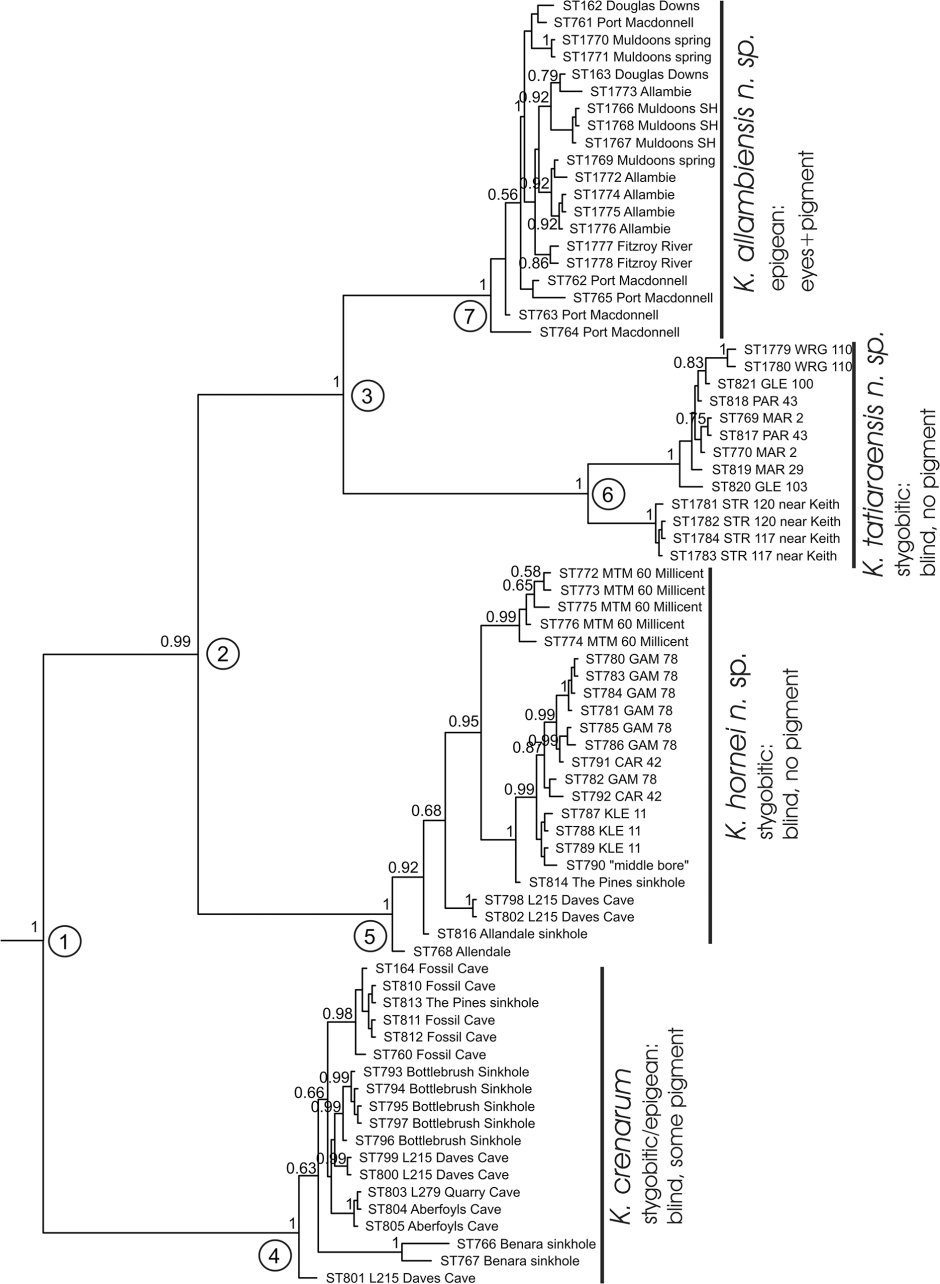
Bayesian phylogenetic tree estimated using MrBayes. Node support values (posterior probabilities) larger than 0.50 are shown at the nodes. Nodes marked with numbers in circles refer to those used in Table 4.

A 658 bp fragment of the COI gene was sequenced from 75 specimens collected from 27 localities (Table 1). Bayesian phylogenetic analyses using MrBayes clearly indicate four well separated lineages (Fig 2) that we consider represent four different species, among which three species are recognised as new. The uncorrected pairwise sequence divergence analysed using PAUP* among these species varied from 10.3–17.7%, while the maximum *intra*-specific pairwise sequence divergence measured for the four species ranged from 2.9 to 6.7% (Table 2). Amino-acid translations indicate that these species have a number of fixed amino-acid substitutions that are diagnostic for each species (Table 3). Results of Bayesian analyses show that the phylogenetic relationships between the species are strongly supported (pp>0.99: Fig 2). *Koonunga crenarum* appears as the sister species of the remaining three species (*K*. *allambiensis* sp. nov., *K*. *tatiaraensis* sp. nov., and *K*. *hornei* sp. nov.). Some of the species (eg. *K*. *tatiaraensis and allambiensis*) show well supported (pp = 1: Fig 2) phylogenetic structure which relates to geographical isolation. However, there were no obvious morphological differences among these specimens to warrant recognition of additional species. Estimates of the divergence times of the nodes in the phylogenetic tree show speciation times of 10.7–22.3 [5.38–33.799 (95% interval)] Mya, while haplotype divergence times of the species varied from 1.13–2.70 [0.29–5.47 (95% interval)] Mya (Table 4).

**Table 1:** Data on sampling localities, DNA extractions, SAMA collection numbers and GenBank accession numbers. An asterix at a SAMA C-number indicates that the specimen is a DNA-voucher. Holotypes are incated in bold and underlined. Locality numbers refer to Fig 1.

	DNA extraction no.	Coll. no.	SAMA	M	F	juv.		locality	feature	dec latitude	dec longitude	collecting date	GenBank
*Koonunga crenarum*													
	**ST760**	RL013	C8393*	0	1	0	1	Fossil Cave, SA	sinkhole	-37.73195	140.53094	1-Sep-04	KR131636
	**ST766-767**	RL017	C8394*	0	0	2	2	Benara sinkhole, SA	sinkhole	-37.85323	140.71113	02-Sep-04	KR131637-8
	**ST793-797**	RL1185	C8395	0	4	4	3	Bottlebrush sinkhole, SA	sinkhole	-37.96951	140.85690	02-Dec-08	KR131639-43
	**ST799-801**	RL1190	C8396	0	4	0	4	L215 Daves Cave, SA	cave	-37.96675	140.58894	03-Dec-08	KR131644-6
	**ST803**	RL1204	C8397	0	0	1	5	L279 Quarry Cave, SA	cave	-37.92204	140.57876	04-Dec-08	KR131647
	**ST804-5**	RL1207	C8398	1	1	0	6	Aberfoyls' Cave, SA	cave	-37.91819	140.57891	04-Dec-08	KR131648-9
	**ST164-5,ST810-2**	RL156					1	Fossil Cave, SA	sinkhole	-37.73195	140.53094	25-Jun-05	KR131650-2
	**ST813**	RL951	C8399	0	1	1	7	The Pines sinkhole, SA	sinkhole	-37.75521	140.54637	21-Jan-08	KR131653
*Koonunga allambiensis*													
	**ST761-765**	RL003	C8400	3	2	0	8	Port MacDonnell, SA	pastoral well	-38.05451	140.76014	31-Aug-04	KR131705-9
	**ST162-3**	RL151	C8401	0	0	2	9	Douglas Downs, SA	spring fed wetland	-37.98431	140.59487	03-Sep-04	KR131690-1
		RL1683	C8402	5	0	0	10	Allambie, VIC	pastoral well	-38.03628	141.82909	22-Mar-11	
	**ST1772**	RL1683	C8403*	1	0	0		Allambie, VIC	pastoral well	-38.03628	141.82909	22-Mar-11	KR131698
	**ST1773**	RL1683	C8404*	0	1	0		Allambie, VIC	pastoral well	-38.03628	141.82909	22-Mar-11	KR131699
		RL1686	**C8443**	1	0	0	10	Allambie, VIC	sinkhole	-38.03253	141.84837	22-Mar-11	
		RL1686	C8405	4	12	2	11	Allambie, VIC	sinkhole	-38.03253	141.84837	23-Mar-11	
	**ST1774**	RL1686		0	0	1		Allambie, VIC	sinkhole	-38.03253	141.84837	22-Mar-11	KR131700
	**ST1775**	RL1686	C8406*	0	1	0		Allambie, VIC	sinkhole	-38.03253	141.84837	22-Mar-11	KR131701
	**ST1776**	RL1686	C8407*	1	0	0		Allambie, VIC	sinkhole	-38.03253	141.84837	22-Mar-11	KR131692
		RL1690	C8408	2	5	1	11	Muldoons, VIC	spring	-38.08231	141.78772	23-Mar-11	
	**ST1769**	RL1690	C8409*	0	1	0		Muldoons, VIC	spring	-38.08231	141.78772	23-Mar-11	KR131694
	**ST1770**	RL1690		0	0	1		Muldoons, VIC	spring	-38.08231	141.78772	23-Mar-11	KR131696
	**ST1771**	RL1690		0	0	1		Muldoons, VIC	spring	-38.08231	141.78772	23-Mar-11	KR131697
		RL1695	C8410	3	8	0	11	Muldoons, near sinkhole, VIC	in lava flow crack	-38.08093	141.79311	23-Mar-11	
	**ST1766**	RL1695	C8411*	0	1	0		Muldoons, near sinkhole, VIC	in lava flow crack	-38.08093	141.79311	23-Mar-11	KR131702
	**ST1767**	RL1695	C8412*	1	0	0		Muldoons, near sinkhole, VIC	in lava flow crack	-38.08093	141.79311	23-Mar-11	KR131693
	**ST1768**	RL1695	C8413*	0	1	0		Muldoons, near sinkhole, VIC	in lava flow crack	-38.08093	141.79311	23-Mar-11	KR131694
		RL1706	C8414	0	1	0	12	Tyrendarra, Fitzroy Rv, VIC	surface water	-38.19827	141.75957	24-Mar-11	
	**ST1777**	RL1706	C8415*	0	1	0		Tyrendarra, Fitzroy Rv, VIC	surface water	-38.19827	141.75957	24-Mar-11	KR131703
	**ST1778**	RL1706	C8416*	0	1	0		Tyrendarra, Fitzroy Rv, VIC	surface water	-38.19827	141.75957	24-Mar-11	KR131704
		RL1689	C8417	0	0	8		Muldoons, spring, VIC	spring	-38.08231	141.78772	23-Mar-11	
		RL1691	C8418	2	1	6		Muldoons, spring, VIC	spring	-38.08231	141.78772	23-Mar-11	
*Koonunga tatiaraensis*													
	**ST769-771**	RL1074	C8419	1	2	0	13	MAR 2, SA	observation bore	-36.60861	140.38614	21-Jul-08	KR131683-4
		RL2138	C8420	0	2	0	14	WRG110 near Keith, SA	observation bore	-36.29325	140.59500	04-May-12	
	**ST1779**	RL2138	C8421*	0	1	0		WRG110 near Keith, SA	observation bore	-36.29325	140.59500	04-May-12	KR131677
	**ST1780**	RL2138	C8422*	1	0	0		WRG110 near Keith, SA	observation bore	-36.29325	140.59500	04-May-12	KR131678
	**ST817-8**	RL979	C8423	0	1	0	15	PAR 43, SA	observation bore	-36.56081	140.45334	28-Apr-08	KR131685-6
	**ST819**	RL984	C8424	0	1	0	16	MAR 29, SA	observation bore	-36.56893	140.40433	28-Apr-08	KR131687
	**ST820**	RL985	C8425	1	0	0	17	GLE 103, SA	observation bore	-36.61239	140.50965	28-Apr-08	KR131688
	**ST821**	RL989	C8426	0	1	0	18	GLE 100, SA	observation bore	-36.67100	140.55858	28-Apr-08	KR131689
	**ST1781-2**	RL2128	C8427	1	1	1	19	STR120 near Keith, SA	observation bore	-36.11148	140.31156	02-May-12	KR131679-80
	**ST1783-4**	RL2132	C8428	0	2	0	20	STR117 near Keith, SA	observation bore	-36.17438	140.39802	02-May-12	KR131681-2
		RL2133	C8429	1	1	0		STR117 near Keith, SA	observation bore	-36.17438	140.39802	02-May-12	
		RL2141	C8430	1	0	0		WRG109 near Keith, SA	observation bore	-36.2700	140.4572	4-May-12	
		RL2143	C8431	1	0	0		STR113 near Keith, SA	observation bore	-36.2204	140.3825	4-May-12	
*Koonunga hornei*													
	**ST787-789**	RL1079	C8432	0	2	1	21	KLE 11, SA	observation bore	-37.20127	140.76815	23-Jul-08	KR131667-9
	**ST790**	RL1087	C8433	0	1	0	22	"middle bore", SA	observation bore	-37.28860	140.83274	23-Jul-08	KR131670
	**ST772-776**	RL1089	C8434	2	2	1	23	MTM 60, SA	observation bore	-37.57350	140.32042	04-May-12	KR131655-9
		RL1089	C8435*	1	0	0		MTM 60, SA	observation bore	-37.57350	140.32042	04-May-12	
	**ST791-2**	RL1180		0	0	1	24	CAR 42, SA	observation bore	-37.92097	140.91146	02-Dec-08	KR131671-2
	**ST780-6**	RL871	C8436	3	1	2	25	GAM 78, SA	observation bore	-37.89655	140.92252	16-Oct-07	KR131660-6
	**ST166,ST768**	RL028	C8437				26	Allandale, SA	observation bore	-38.00463	140.70677	02-Sep-04	KR131654
	**ST798,802**	RL1190	C8438				4	L215 Daves Cave, SA	cave	-37.96675	140.58894	03-Dec-08	KR131673-4
	**ST816**	RL962	C8439	0	1	0	27	Allandale Sinkhole, SA	cave	-38.00509	140.70840	23-Jan-08	KR131676
	**ST814**	RL951	C8399*	0	1	0	7	The Pines sinkhole, SA	sinkhole	-37.75521	140.54637	21-Jan-08	KR131675
		RL994	C8440	0	0	1		GAM 78, SA	observation bore	-37.89655	140.92252	30-Apr-08	
		RL947	C8441	1	0	1		GAM 78, SA	observation bore	-37.89655	140.92252	21-Jan-08	

**Table 2:** Uncorrected pairwise distances, minimum and maximum *intra*- (diagonal) and *inter-* specific values.

	*K*. *alambieensis*		*K*. *tatiaraensis*		*K*. *hornei*		*K*. *crenarum*	
species	min	max	min	max	min	max	min	max
*K*. *alambieensis sp*. *nov*.	0	0.029						
*K*. *tatiaraensis* sp. nov.	0.103	0.129	0	0.052				
*K*. *hornei* sp. nov.	0.105	0.155	0.119	0.142	0	0.04		
*K*. *crenarum*	0.126	0.167	0.142	0.177	0.118	0.173	0	0.067

**Table 3:** Overview of fixed amino acid substitutions relative to *K*. *crenarum*. Highlighted amino acids show species specific sites. Amino acid positions are relative to the first amino acid upstream of the forward primer, and relative to *K*. *crenarum* (a dash indicates same amino-acid as in *K*. *crenarum*).

amino-acid site							1	1	1	1	1	1	1	1
	1	1	2	3	4	9	0	1	1	2	5	6	6	6
Species	2	5	3	0	8	4	1	8	9	0	2	1	5	9
*K*. *crenarum*	M	T	T	S	I	T	M	A	G	I	I	T	I	A
*K*. *hornei* sp. nov.	-	-	A	-	V	V	L	S	S	V	-	K	-	V
*K*. *tatiaraensis* sp. nov	L	S	A	N	-	V	-	-	-	V	V	S	V	V
*K*. *allambiensis* sp. nov.	L	-	A	-	-	V	-	-	-	-	-	K	-	V

**Table 4:** Estimates of divergence times from BEAST analyses using a mean uncorrelated lognormal clock rate prior of 0.0105/My. The node numbers refer to Fig 2.

Node	mean age	95% interval
node 1 (ingroup)	22.29	12.66–33.78
node 2	16.07	9.00–24.36
node 3	10.07	5.38–15.60
node 4 (*K*. *crenarum*)	2.49	1.07–5.47
node 5 (*K*. *hornei* sp. nov.)	2.52	0.89–4.73
node 6 (*K*. *tatiaraensis* sp. nov.)	2.70	1.09–4.75
node 7 (*K*. *allambiensis* sp. nov.)	1.13	0.29–2.25

### Geographical distribution of the species

There is interesting geographical and ecological separation of the species. *K*. *tatiaraensis* sp. nov. has only been found in groundwater monitoring bores in the northern part of the Mount Gambier aquifer near Keith and Padthaway, while its sister species, *K*. *allambiensis* sp. nov. occurs in coastal surface waters, such as spring fed swamps and rivers between Douglas Downs, SA and the Tyrendera River, VIC. (Fig 1). The distribution of *K*. *crenarum*, the largest species, seems to be restricted to the surface water of the many cenotes around Mount Gambier, however numerous specimens have been collected by cave divers at depths up to 40 m and far from the sinkhole entrances [16]. The distribution of the third new species, *K*. *hornei* sp. nov., partly overlaps with *K*. *crenarum*, but it also occurs as far north as Penola. Contrary to *K*. *crenarum*, this species is mainly collected from groundwater monitoring bores, but occasionally has been found sympatric with *K*. *crenarum* in some caves, e.g. The Pines sinkhole and L215 Dave’s Cave (Fig 1, Table 1).

### Key to the currently known species of *Koonunga*

Note: this key is useful to identify the species from the area depicted in Fig 1 with the addition of *K*. *cursor* known from the Melbourne area. It is very likely that southeastern Australia (Victoria, Tasmania and southern New South Wales) contains additional undescribed species.

**1.** Eye absent (no eye pigment) or undefined (with diffused pigment present across the anterior margin of the head); sternal process in males without posterior projection (Figs 10B, 14B) .......... **2**
—Defined eye present (with concentrated spot of pigment); sternal process in males with posterior projection (Fig 6B) .................................................................... **4**
**2.**Adult body size exceeding 14 mm; antennule length 3/4 body length .......................... ***K*. *crenarum***
—Adult body size less than 10 mm; antennule length 1/2 body length ............................. **3**
**3.** Adult body size 5–10 mm; robust setae on last pleonite flanked with a smaller seta medially (Fig 15B); male with sensory scale on antennule ............................ ***K*. *hornei* sp. nov.**
—Adult body size less than 5 mm; robust setae on last pleonite flanked with a smaller seta laterally (Fig 11B); male without sensory scale on antennule ....................... ***K*. *tatiaraensis* sp. nov.**
**4.** Antennule length 1/2 body length; last pleonite with a single robust seta dorsolaterally
...................................................................................... ***K*. *cursor***
—Antennule length 2/5 body length; last pleonite with two robust setae dorsolaterally, flanked by smaller ones on each side (Fig 7B) ....................................... ***K***. ***allambiensis* sp. nov.**

### Systematics

**Syncarida Packard, 1885**[38]

**Anaspidacea Calman, 1904**[1]

**Koonungidae Sayce, 1907**[8]

**Diagnosis (Modified after Poore [10]):** Rostrum broad. Eyes sessile or absent. Mandible with palp. Maxilla 1 with palp. Maxilliped (thoracopod 1) of 7 articles, with tubular exopod and epipod. Pereonite 1 fused with the head, pereonites 2–8 free, pereonites of similar lengths to pleonites. Pleonites 1–6 free, pleonite 6 without row of long spines along posterior margin. Pleopods, with multiarticulate exopod; without endopod except in adult males where pleopods 1–2 endopodites modified to form complex copulatory structures (petasma), directed anteriorly against sternal surface between the last pair of pereopods. Uropods with rami of 1 article. Telson subtriangular, forming tail-fan with uropods. Seminal receptacle present in female.

**Remarks:** The publication date for *Koonunga* and Koonungidae is often given as 1908, based on the paper published in the Annals but, but this paper was a reproduction of the 1907 paper originally published in the Victorian Naturalist. Thus, the correct date of publication of the names is 1907. Family diagnoses in Poore [10] stated that the differences between the Anaspididae and the Koonungidae included “thoracopod 1 of 8 articles and forming maxilliped in Anaspididae” and “…of 7 articles and not forming a maxilliped” in Koonungidae, and antenna with scaphocerite in Anaspididae and without scaphocerite in Koonungidae. We find that thoracopod 1 in taxa of both families is similar, of 7 articles and forms a maxilliped.

***Koonunga* Sayce, 1907** [8]

**Composition:** *K*. *allambiensis* sp. nov., *K*. *crenarum*, *K*. *cursor*, *K*. *hornei* sp. nov., *K*. *tatiaraensis* sp. nov.

**Diagnosis (After Sayce [8]; Zeidler, 1985[16]):** Head (including fused pereonite 1) about equal in length to following 2–3 pereonites, with anterolateral incision above attachment of antenna, dorsally with pronounced short mid-lateral transverse sulcus. Antennule in males with or without oval-shaped sensory organ on the second article of the outer flagellum (lacking only in *K*. *tatiarensis* sp. nov.). Maxilliped (thoracopod 1) and pereopods 1–5 (thoracopods 2–6) with exopod, pereopods 6–7 (thoracopods 7–8) without exopod. All pereopods flexed between merus and carpus and in backward position except pereopod 6 which flexes inwards and pereopod 7 which flexes forwards. Pleopods 1–2 endopod in adult males modified to form a petasma. Male with median sternal process situated at the base of the second pleopods.

***Koonunga allambiensis* Leijs & King**, **sp**. **nov**.

(Figs 3–7)

**Fig 3:**
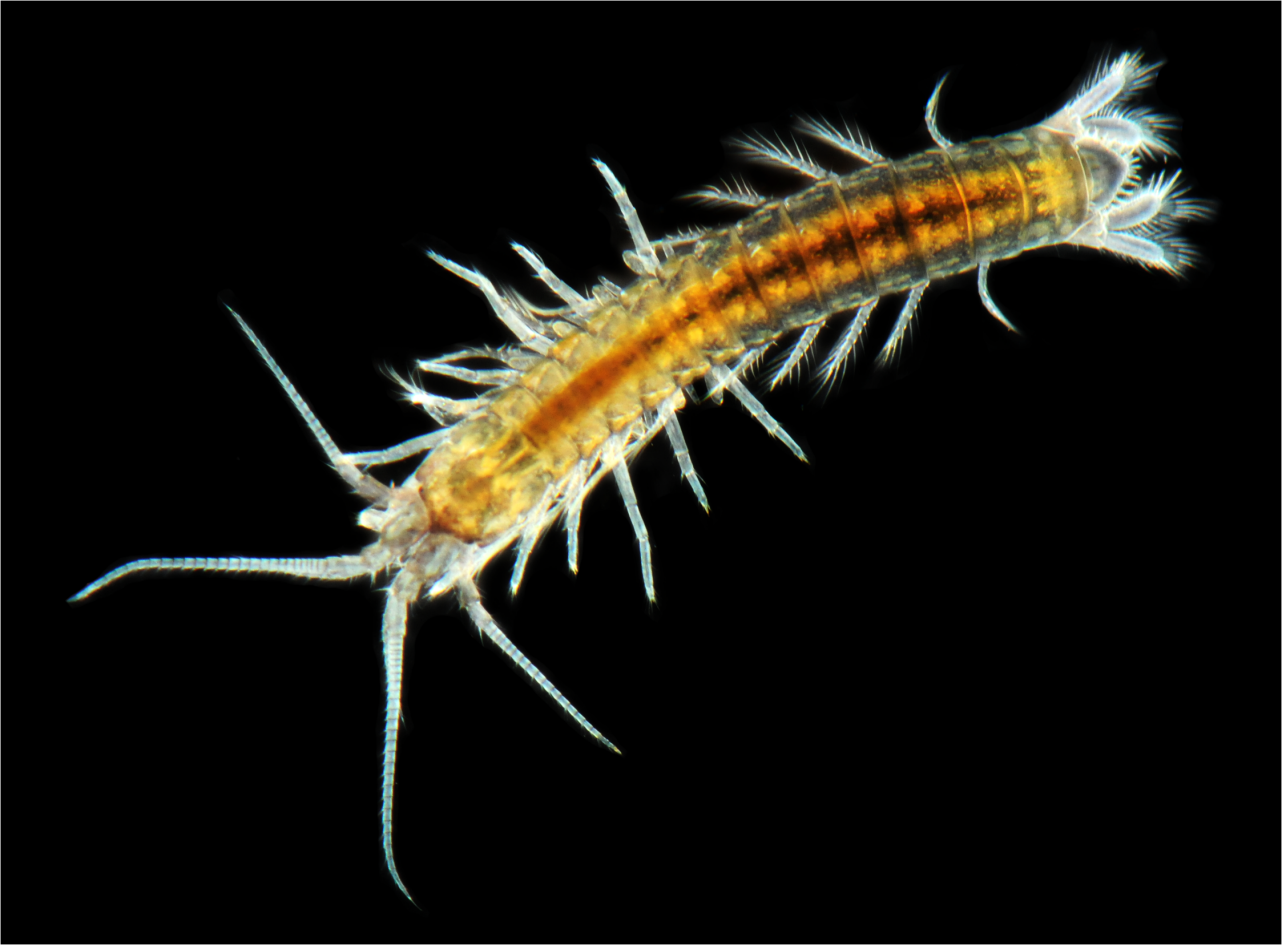
*Koonunga allambiensis* sp. nov. habitus (Photo Julian Finn, Victorian Museum)

**Fig 4:**
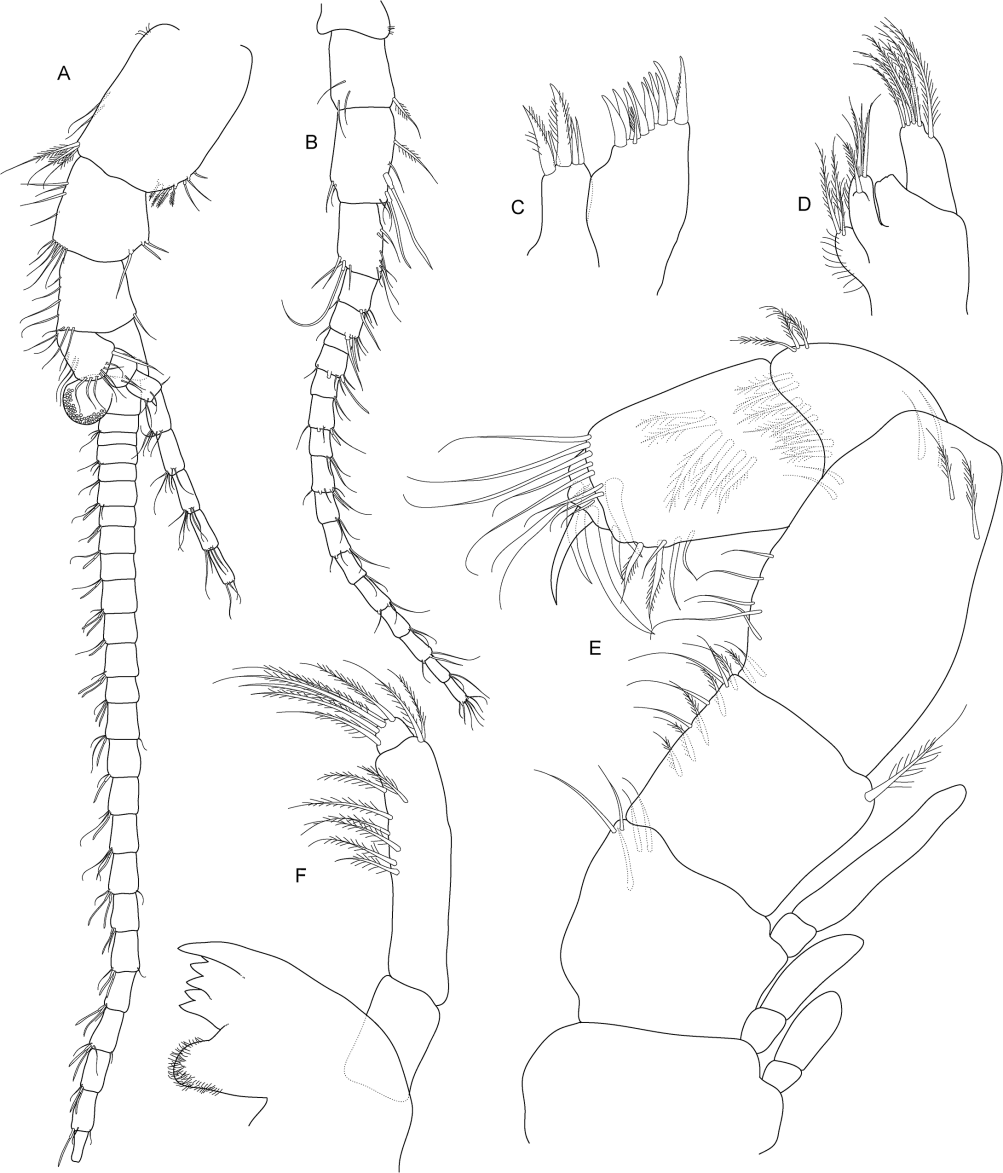
*Koonunga allambiensis* sp. nov., holotype male, 5.95 mm, SAMA C8443: A, antennule; B, antenna; C, maxilla 1; D, maxilla 2; E, maxilliped; F, mandible.

**Fig 5:**
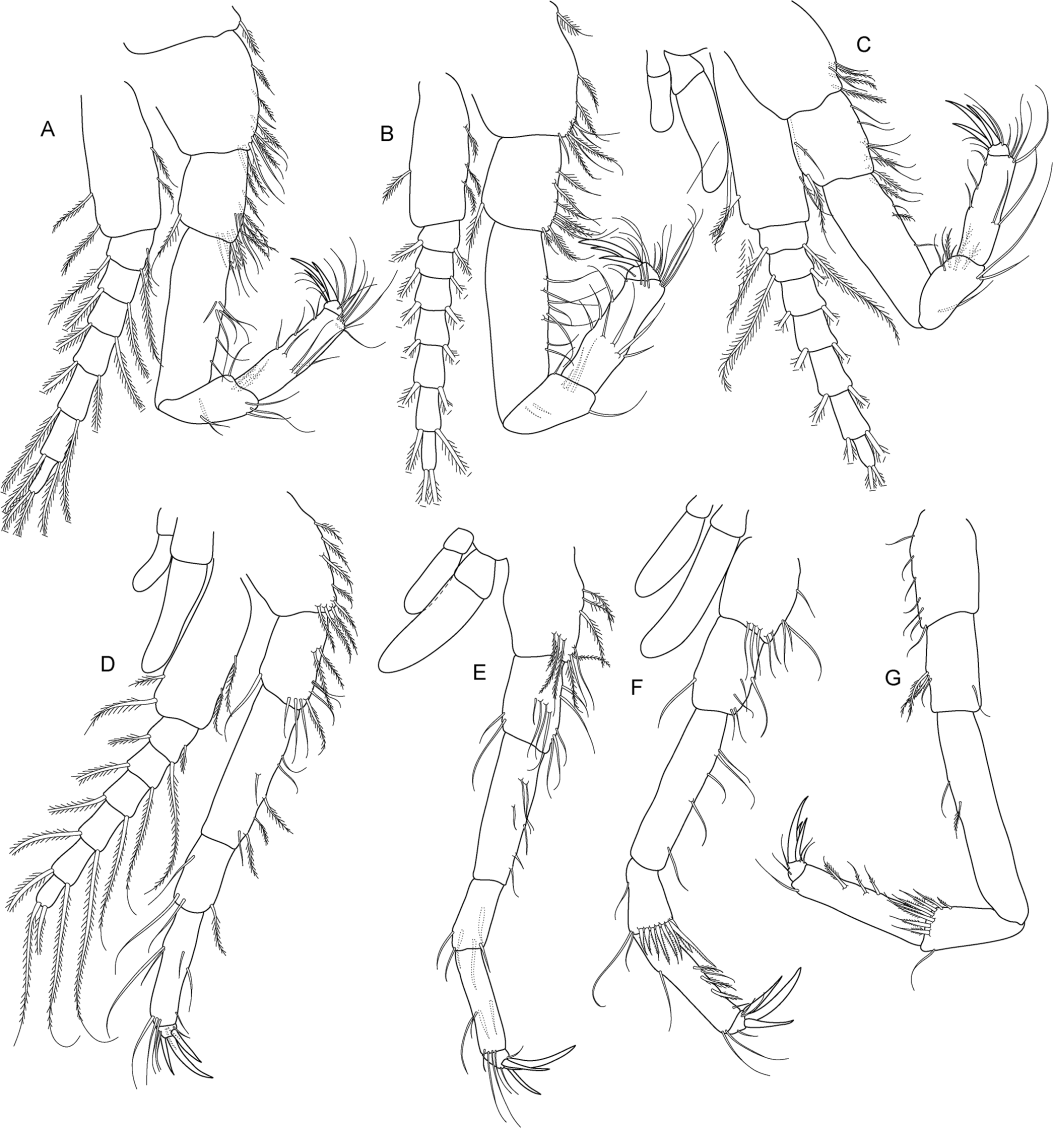
*Koonunga allambiensis* sp. nov., holotype male, 5.95 mm, SAMA C8443: A, pereopod 1; B, pereopod 2; C, pereopod 3; D, pereopod 4; E, pereopod 5; F, pereopod 6; G, pereopod 7.

**Fig 6:**
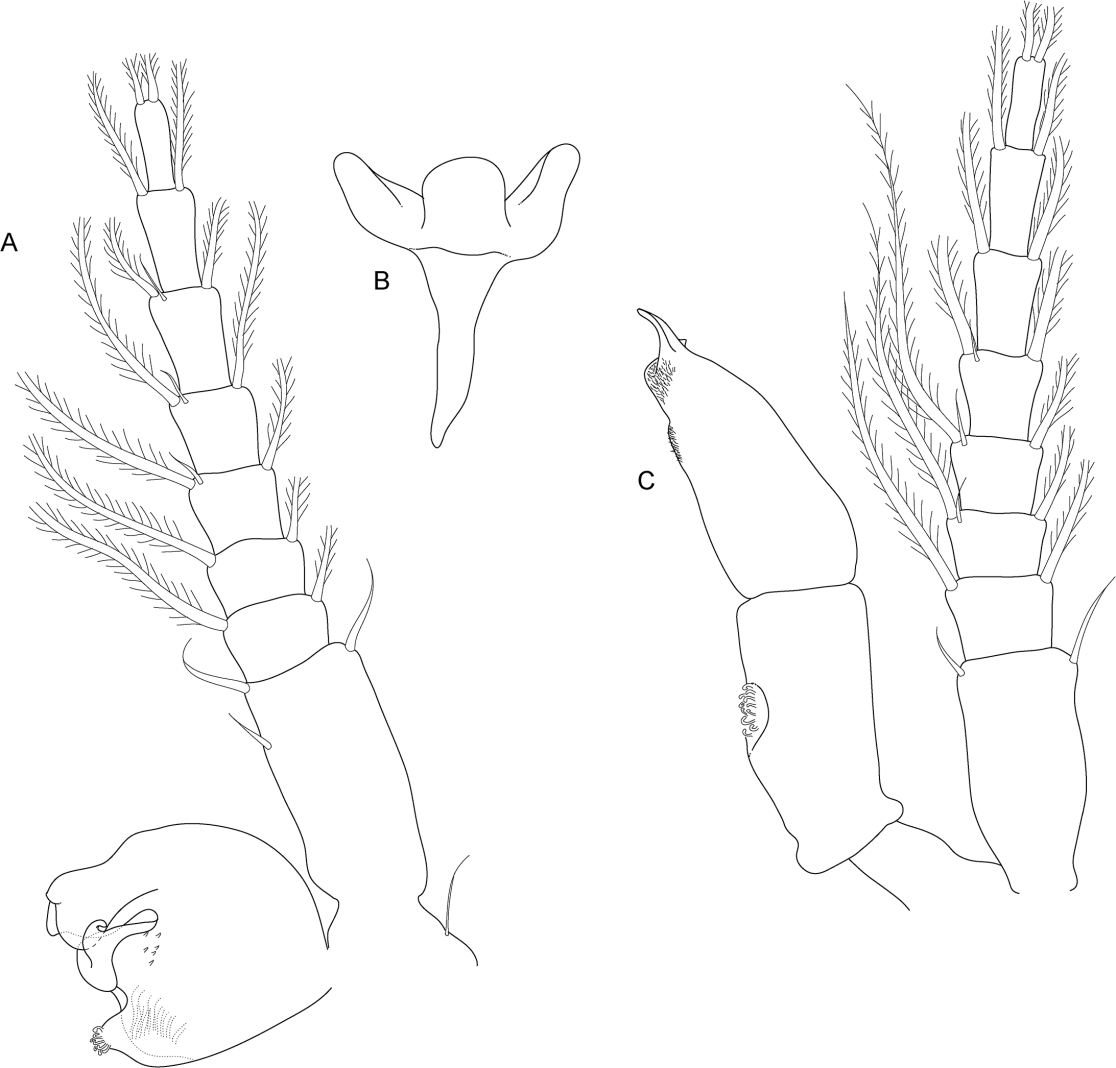
*Koonunga allambiensis* sp. nov., holotype male, 5.95 mm, SAMA C8443: A, pleopod 1; B, endopod (petasma) ventral view; C, pleopod 2

**Fig 7:**
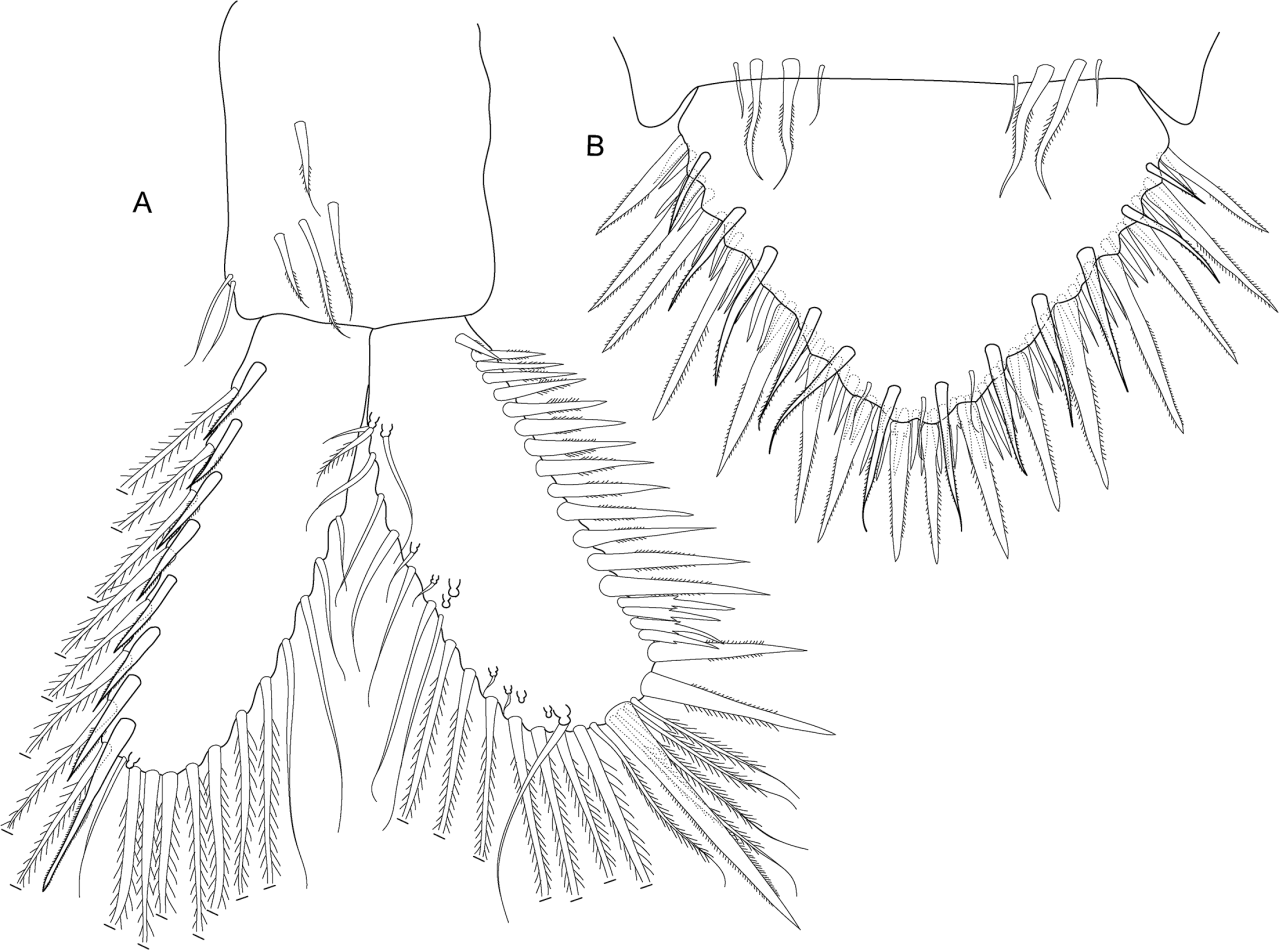
*Koonunga allambiensis* sp. nov., holotype male, 5.95 mm, SAMA C8443: A, uropod; B, telson.

LSID urn:lsid:zoobank.org:act:44FDF054-DE95-4175-8A03-545962296EBE

**Material examined:** *Holotype*. SAMA C8443, male, 5.95 mm, Allambie, VIC, sinkhole, 22-Mar-2011, -38.03253°S, 141.84837°E, coll. R. Leijs. *Paratypes*. SAMA C8405, SAMA 8406, 4 males, 13 females, 2 juveniles, collected with holotype.

**Additional material examined:** see Table 1.

**Molecular diagnosis:** Molecular diagnostic characters: five fixed amino acid differences relative to *K*. *crenarum*. Amino acid positions are relative to the first amino acid downstream of the M414 primer site: L at position 12; A at position 23, V at positions 94 and169, and K at position 161. (Table 3).

**Description:** Holotype male (SAMA C8443)

Length 5.95 mm. Antennule length 2.41 mm. Antenna length 1.53 mm. Head length 0.93 mm. Pereon length 2.39 mm. Pleon length 2.29 mm. Telson length 0.34 mm. Pleon length (excluding telson) 0.39mm.

Head rectangular, with short pointed rostrum. Defined eye present, with pigment.

Antennule (Fig 4A) length 0.4 times body length; peduncle with 3 articles, basal article as long as following two combined, articles 1 and 2 outer margins with small plumose setae, simple setae and type 5 setae, inner margins with 5–8 setae, of which distal setae most robust; outer flagellum with 25 articles, about 2.8 times the length of inner flagellum, sensory organ present on article 2, with aesthetascs present on all articles; inner flagellum with 8 articles.

Antenna (Fig 4B) length slightly more than 0.62 of antennule length; peduncle with 4 articles, basal article very short, articles 2–4 elongate and rectangular shaped, article 3 longest, articles distally with 3–4 type 5 setae; flagellum with 16 articles, type 5 setae on every second article.

Mandible (Fig 4F) palp with 3 articles; article 2 greatly enlarged, about 3.3 times length article 1, with 7 plumose setae along inner margin; article 3 small, rounded, with plumose setae apically. Base of mandible stout, ending in well-developed molar and incisor process. Molar process with small grinding surface surrounded by numerous spiniform setae. Incisor process of left mandible with five denticles. No evidence of secondary cutting plate or spine row.

First maxilla (Fig 4C) two lobed; outer lobe with small one segmented palp (not illustrated as it was missing on the animal) with three robust plumose setae, extremity of outer lobe obliquely truncated with 10 setulate robust setae, some stouter than others and 1 smaller setulate seta near outer (aboral) surface; Inner lobe about ½ width of outer lobe with 2 robust setulate setae near inner (oral) surface surrounded by 3 smaller setulate setae.

Second maxilla (Fig 4D) smaller than first maxilla, consisting of four lobes, inner lobe smallest with the others increasing successively in length and width; inner lobe covered with fine simple setae, with 3 plumose setae distally; outer lobes with more numerous (3–6) slightly longer setae (third lobe damaged).

Maxilliped (Fig 4E) (thoracopod 1) stout, of 7 articles flexed posteriorly between articles 4 and 5; article 1 (coxa) shorter and wider than following articles with 2 epipodites near outer aboral corner; article 2 (basis) with exopodite of 2 articles resembling epipodites, exopodite reaching past article 3, aboral margin with several simple setae near distal oral margin; article 3 (ischium) slightly expanded on outer distal corner, slightly wider and longer than article 2, with several long fine plumose setae on inner margin and a single long plumose seta on outer aboral corner; article 4 (merus) inflated proximally, longer than any other segment, only slightly narrower than article 1; article 5 (carpus) small, with row of long setae along distal, inner margin for inner half and on oral and aboral outer distal margin; article 6 (propodus) robust, slightly shorter than article 4, with two oblique rows of setulate setae on oral surface and row of long setae aboral distal corner; article 7 (dactylus) small, rounded, with 5 strong claw-like robust setae (two large, three small) and long setae near inner margin.

Pereopods 1–7 (thoracopods 2–8) (Fig 5A–5G) similar in structure to maxilliped, progressively becoming more slender with all articles more elongate. Pereopods 1–6 slightly shorter than maxilliped, pereopods 5 and 6 shortest overall, pereopod 7 longest overall. Pereopods 1–6 coxa with two unequal epipodites as in maxilliped. Pereopods 1–5 bases with multi-segmented exopodite consisting of large basal segment reaching well past ischium (exopodite not illustrated in Fig 4E). Pereopod 6 basis without exopodite. Pereopod 7 without epipodite or exopodite. Pereopods 6–7 carpus distal margin with a row of strong plumose setae. Pereopods 1–7 dactylus with 3 claw-like robust setae, similar to maxilliped.

Pleopods 1–2 (Fig 6A and 6C) with endopodites modified to form complex copulatory structure (petasma). Pleopod 1 endopodite of one article and joined by coupling hooks on a lobe on the inner margin (Fig 6A). Pleopod 2 endopodite of two articles of about equal length, each as long as endopodite of pleopod 1; basal article with coupling hooks on inner margin on small pad near at mid length (Fig 6C); distal article apically pointed, hollowed out distally, inner margin forming a concave depression directed towards body. Pleopods 3–5 of similar structure (not illustrated), lacking endopodites but with multi-segmented exopodites consisting of basal article and flagellum of seven articles, each bearing two long plumose setae. Sternal process (Fig 6B) anterior median lobe broadly rounded, posterior median lobe long and spiniform, lateral lobes longer than median lobe.

Uropod with peduncle stout (Fig 7A), rectangular about 1.6 times as long as wide, and slightly shorter than rami, with 4 strong setulate setae on dorsal surface and near outer margin; outer ramus slightly longer than inner ramus, with around 20 long plumose setae along inner and outer margins and row of 8 short strong upturned setulate setae near outer dorsal margin; inner ramus with around 18 long plumose setae along outer and proximal margin; inner proximal margin with three dorsally directed terminal robust setulate setae, the two smaller setae ~2/3 length of terminal seta; dorsal inner margin with row of 14 strong upturned curved setulate setae for about proximal 2/3 length, steadily increasing in size followed by a row of four smaller tridentate setae ending at first terminal seta; dorsal outer margin on inner ramus with row of often paired type 5 setae.

Telson (Fig 7B) triangular with narrow rounded apex, length (excluding spines) slightly less than width, margins with three arrangements of spines (in order dorsal to ventral): 12 posterodorsally directed robust setulate setae interspersed with simple setae medially, 16 posteroventrally directed robust setulate setae, ~ 46 posteriorly directed short setae about 1/3 in length of posteroventrally directed setae with two pairs of 3 tridentate setae subapically.

**Distribution:** South-east South Australia and south-west Victoria (Fig 1), coastal, in groundwater fed marshlands and creeks.

**Etymology:** Named after the type locality: Allambie, Victoria.

**Remarks:** *K*. *allambiensis* sp. nov. is the most easily recognised of the three new species described here by its possession of a defined pigmented eye and male sternal process with a posterior projection (lacking in *K*. *hornei* sp. nov. and *K*. *tatiaraensis* sp. nov.). It can also be distinguished from *K*. *crenarum* by its possession of a defined eye and male sternal process morphology. *K*. *allambiensis* sp. nov. is very similar to *K*. *cursor* in uropod and pleopod morphology, however, it can be distinguished by the telson shape, which is more acute posteriorly, not as rounded as in *K*. *cursor* and in the setation of the last pleonite which possesses 2–3 robust setae dorsolaterally (vs only 1 robust seta in *K*. *cursor*) (see Zeidler (1985) [16] for additional illustrations).

***Koonunga crenarum* Zeidler**, **1985[16]**

***Koonunga crenarum* Zeidler**, **1985: 63–75, Figs 2–8**.

**Fig 8:**
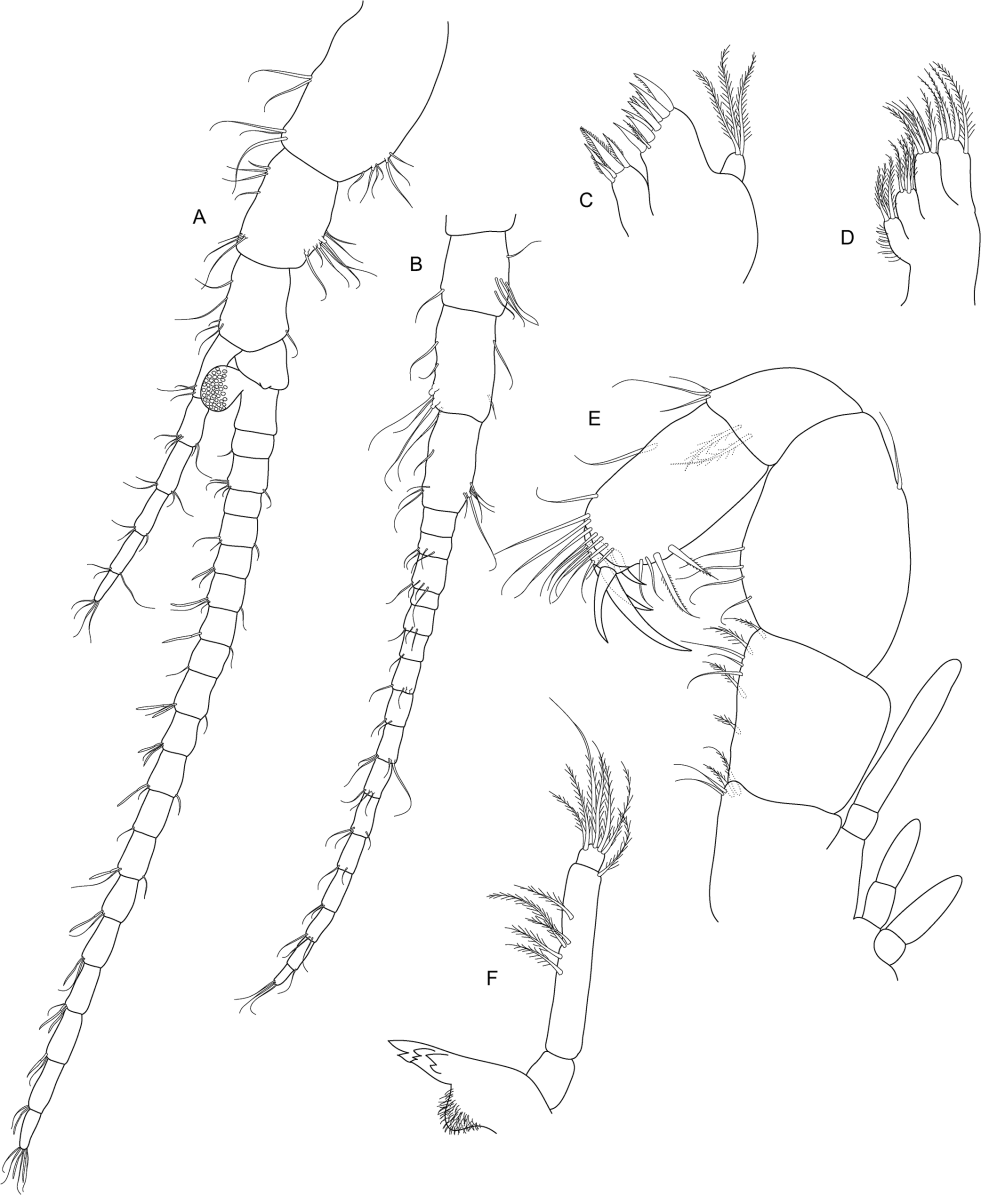
*Koonunga hornei* sp. nov., holotype male, 6.66 mm, SAMA C8435: A, antennule; B, antenna; C, maxilla 1; D, maxilla 2; E, maxilliped; F, mandible.

*Material examined*: SAMA C3989, dissected holotype female, Fossil Cave sinkhole, SA, approx. 3.3 km SE of Tantanoola Caves, 1-Mar-1982, coll. W. Zeidler. SAMA C3990, dissected allotype male, same locality of holotype, 8-Mar-1981, coll. P. Horne. SAMA 3991, paratype female, same locality of holotype, 1-Mar-1982, coll. W. Zeidler. SAMA 3992, paratype male, same locality of holotype, 8-Mar-1981, coll. P. Horne.

*Additional material examined*: see Table 1.

***Koonunga cursor* Sayce**, **1907[8]**

***Koonunga cursor* Sayce**, **1907: 117–120.—Zeidler, 1985 (in part): 73, Figs 5 and 6**.

**Material examined:** NMV J1046, 1 dissected syntype and 1 co-type male (type data as on original labels), Mullum Creek, Ringwood, VIC. SAMA C4016, 6 individuals, Railway drain, Bayswater, VIC, 16-Jul-1980, coll. F.H. Drummond.

***Koonunga hornei* Leijs & King, sp. nov**.

(Figs 8–11)

**Fig 9:**
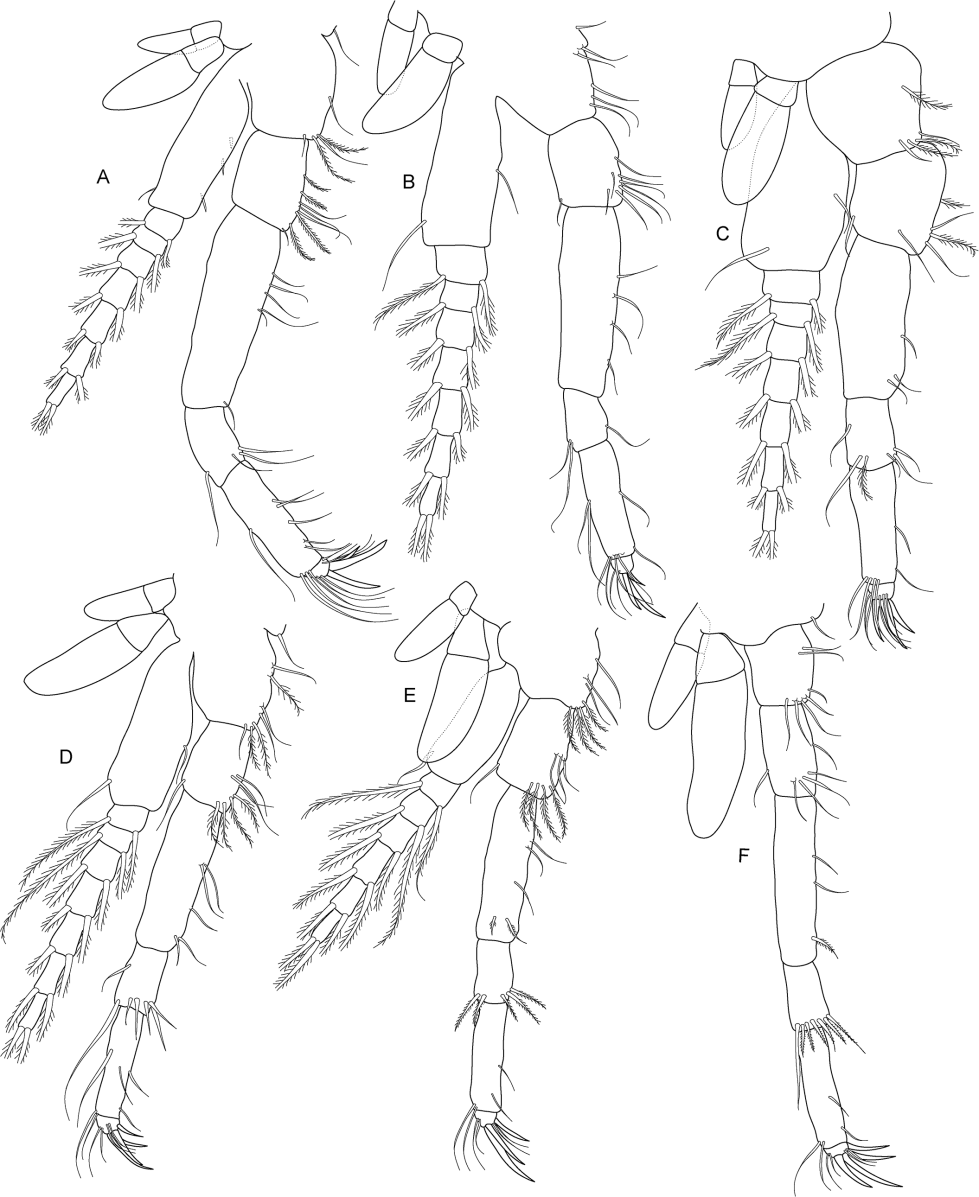
*Koonunga hornei* sp. nov., holotype male, 6.66 mm, SAMA C8435: A, pereopod 1; B, pereopod 2; C, pereopod 3; D, pereopod 4; E, pereopod 5; F, pereopod 6; G, missing.

**Fig 10:**
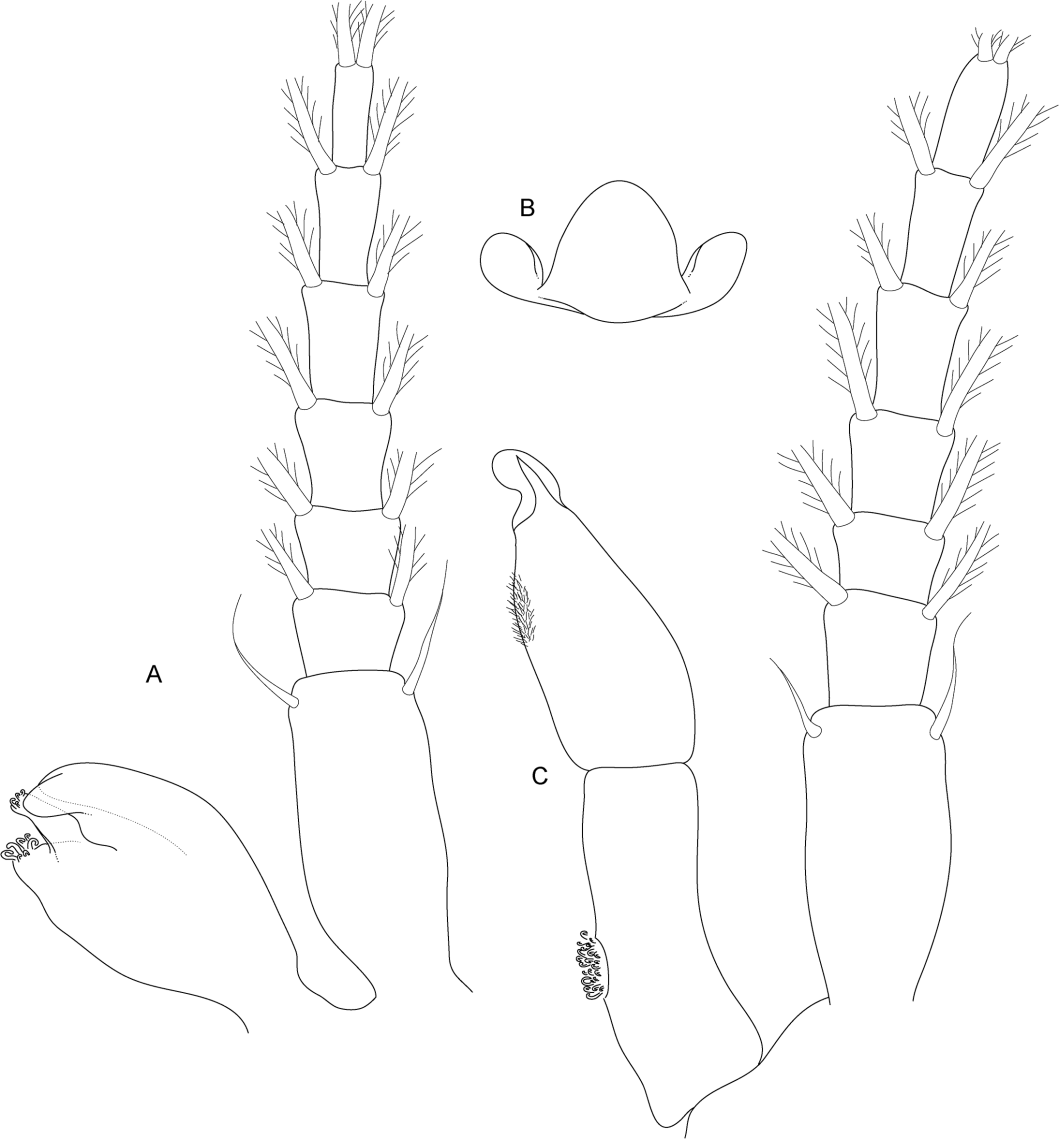
*Koonunga hornei* sp. nov., holotype male, 6.66 mm, SAMA C8435: A, pleopod 1; B, endopod (petasma) ventral view; C, pleopod 2.

**Fig 11:**
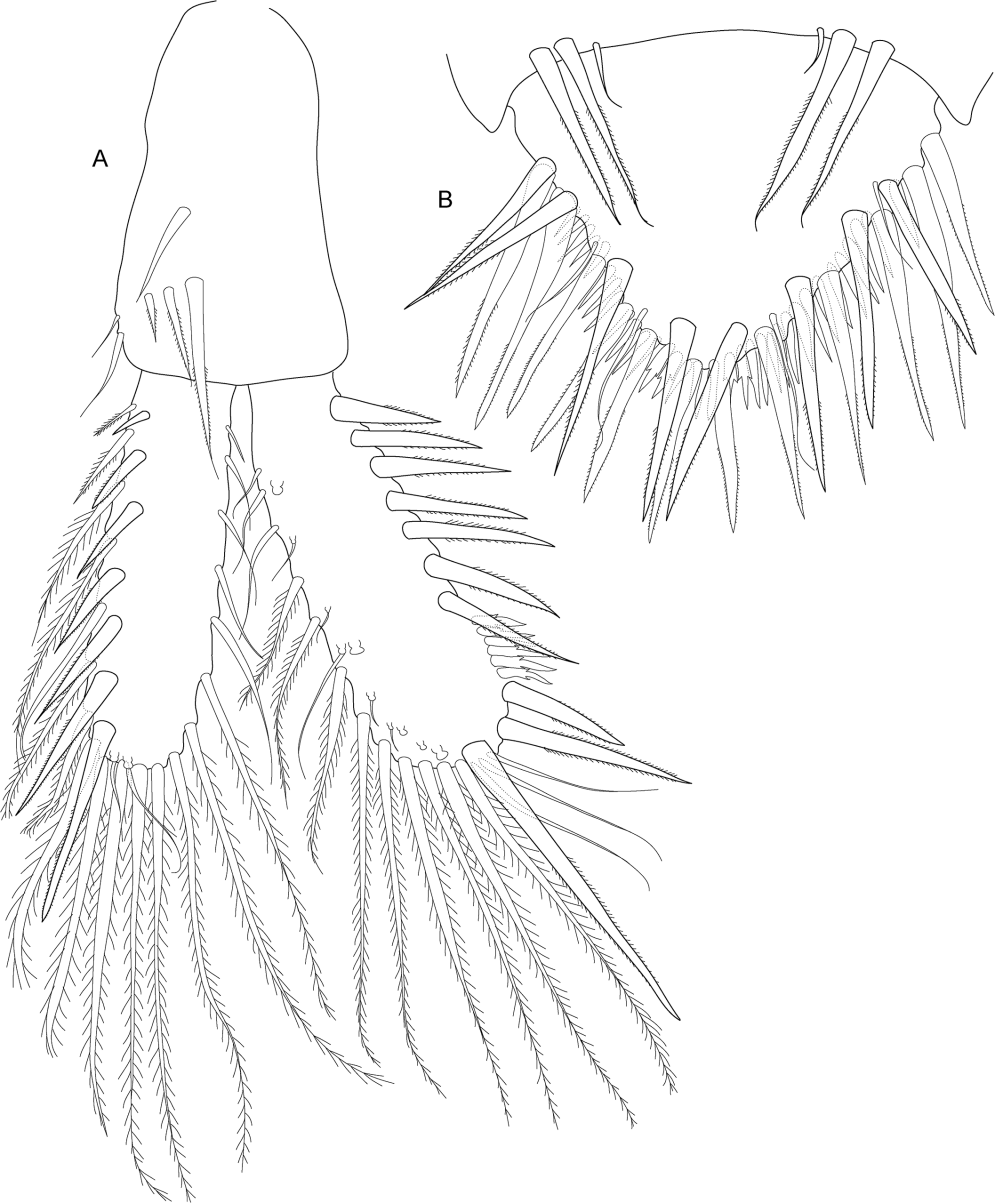
*Koonunga hornei* sp. nov., holotype male, 6.66 mm, SAMA C8435: A, uropod; B, telson.

LSID urn:lsid:zoobank.org:act:2D218816-9AD5-4399-8DD5-72BCB5F6EDFE

**Material examined:** *Holotype*. SAM C8435, male, 6.61 mm, near Millicent, SA, observation bore, 04-May-2012, 37.5735°S, 140.32042°E, coll. R. Leijs, *Paratypes*. SAM C8434, 2 males, 2 females, 1 juvenile, collected with holotype.

**Additional material examined:** see Table 1.

**Molecular diagnosis:** Molecular diagnostic characters: nine fixed amino acid differences relative to *K*. *crenarum*, four are unique for the species. Amino acid positions are relative to the first amino acid downstream of the M414 primer site: A at positions 23; V at position 48; L at position 101; S at positions 118, 119 (Table 3).

**Description:** Holotype male (SAMA C8435)

Length 6.61 mm. Antennule length 3.34 mm. Antenna length 2.1 mm. Head length 0.97 mm. Pereon length 2.61 mm. Pleon length 2.58 mm. Telson length 0.45 mm. Pleon length (excluding telson) 0.37 mm.

Head rectangular, with short pointed rostrum. Defined eyes absent, pigment present and diffuse across the anterior margin of the head.

Antennule (Fig 8A) length 0.5 times body length; peduncle with 3 articles, basal article slightly longer than following two combined, articles 1 and 2 on outer margins with small plumose setae, simple setae and 2–3 type 5 setae, inner margins with 5–8 setae, of which distal setae most robust; outer flagellum with 21 articles, about 2.9 times the length of inner flagellum, sensory organ present on article 2, aesthetascs present on all articles; inner flagellum with 6 articles.

Antenna (Fig 8B) length slightly more than 0.62 of antennule length; peduncle with 4 articles, basal article very short, articles 2–4 elongate and rectangular shaped, article 3 longest, articles distally with 3–4 type 5 setae; flagellum with 16 articles, type 5 setae on almost every second article.

Mandible (Fig 8F) palp with 3 articles; article 2 greatly enlarged, about 4.6 times length article 1, with 5 plumose setae along inner margin; article 3 small, rounded, with plumose setae apically. Base of mandible stout, ending in well-developed molar and incisor process. Molar process with small grinding surface surrounded by numerous spiniform setae. Incisor process of left mandible with six denticles. No evidence of secondary cutting plate or spine row.

First maxilla (Fig 8C) two lobed; outer lobe with small one segmented palp, with three robust plumose setae, extremity of outer lobe obliquely truncated with 9 setulate robust setae, some stouter than others and 1 smaller setulate seta near outer (aboral) surface; inner lobe about ½ width of outer lobe with 2 robust setulate setae near inner (oral) surface surrounded by 2 smaller setulate setae.

Second maxilla (Fig 8D) smaller than first maxilla, consisting of four lobes, inner lobe smallest with the others increasing successively in length and width; inner lobe covered with fine simple setae, with 3 plumose setae distally; outer lobes with more numerous (3–4) similar but little longer setae.

Maxilliped (Fig 8E) (thoracopod 1) stout, of 7 articles flexed posteriorly between articles 4 and 5; article 1 (coxa) shorter and wider than following articles with 2 epipodites near outer aboral corner; article 2 (basis) with exopodite of 2 articles resembling epipodites, exopodite reaching past article 3, aboral margin with several simple setae near distal oral margin; article 3 (ischium) slightly expanded on outer distal corner, slightly wider and longer than article 2, with several long fine plumose setae on inner margin and a single long plumose seta on outer aboral corner; article 4 (merus) inflated proximally, longer than any other segment, only slightly narrower than article 1; article 5 (carpus) small, with row of long setae along distal, inner margin for inner half and on oral and aboral outer distal margin; article 6 (propodus) robust, slightly shorter than article 4, with oblique setulate setae on oral surface and row of long setae aboral distal corner; article 7 (dactylus) small, rounded, with 4 strong claw-like robust setae (one large, three small) and long setae near inner margin.

Pereopods 1–7 (thoracopods 2–8) (Fig 9A–9F) similar in structure to maxilliped, progressively becoming more slender with all articles more elongate. Pereopod 1 slightly longer than maxilliped (longest pereopod), pereopods 2–6 slightly shorter than maxilliped, pereopods 5 and 6 shortest overall. Pereopod 7 not dissected. Pereopods 1–6 coxa with two unequal epipodites as in maxilliped. Pereopods 1–5 bases with multi-segmented exopodite consisting of large basal segment reaching well past ischium. Pereopod 6 basis without exopodite. Pereopod 6 carpus distal margin with a row of strong plumose setae. Pereopods 1–6 dactylus with 3–4 claw-like robust setae, similar to maxilliped.

Pleopods 1–2 (Fig 10A–10C) with endopodites modified to form complex copulatory structure (petasma). Pleopod 1 endopodite of one article and joined by coupling hooks on a lobe on the inner margin (Fig 10A). Pleopod 2 endopodite of two articles of about equal length, each as long as endopodite of pleopod 1; basal article with coupling hooks on inner margin on small pad at less than half length (Fig 10C); distal article apically flattened and rounded, hollowed out distally, inner margin forming a concave depression directed towards body, with tuft of fine setae at mid length. Pleopods 3–5 of similar structure (not illustrated), lacking endopodites but with multi-segmented exopodites consisting of basal article and flagellum of five to six articles, each bearing two long plumose setae. Sternal process (Fig 10B) anterior median lobe dome shaped, no posterior median lobe, lateral lobes shorter than median lobe.

Uropod with peduncle stout (Fig 11A) rectangular, about 1.4 times as long as wide, and slightly shorter than rami, with three robust setulate setae on dorsal surface and near outer margin; outer ramus slightly longer than inner ramus, with around 20 long plumose setae along inner and outer margins and row of seven short strong upturned setae near outer dorsal margin; inner ramus with around 15 long plumose setae along outer and proximal margin; inner proximal margin with three dorsally directed terminal robust setulate setae, the two smaller setae less than ½ length of terminal seta; dorsal inner margin with row of seven strong upturned curved setae for about proximal 2/3 length steadily increasing in size followed by a row of six smaller tridentate setae, ceasing at first terminal seta; dorsal outer margin on inner ramus with row of often paired type 5 setae.

Telson (Fig 11B) triangular, with narrow rounded apex and slightly convex lateral margins, length (excluding spines) slightly less than width, margins with three arrangements of spines (in order dorsal to ventral): eight posterodorsally directed robust setulate setae interspersed with simple setae medially, 12 posteroventrally directed robust setulate setae, 24 posteriorly directed short setae about 1/3 in length of posteroventrally directed setae with two pairs of two tridentate setae sub-apically.

**Variation:** In some individuals a small concentration of pigment is present above the antero-lateral incision.

**Fig 12:**
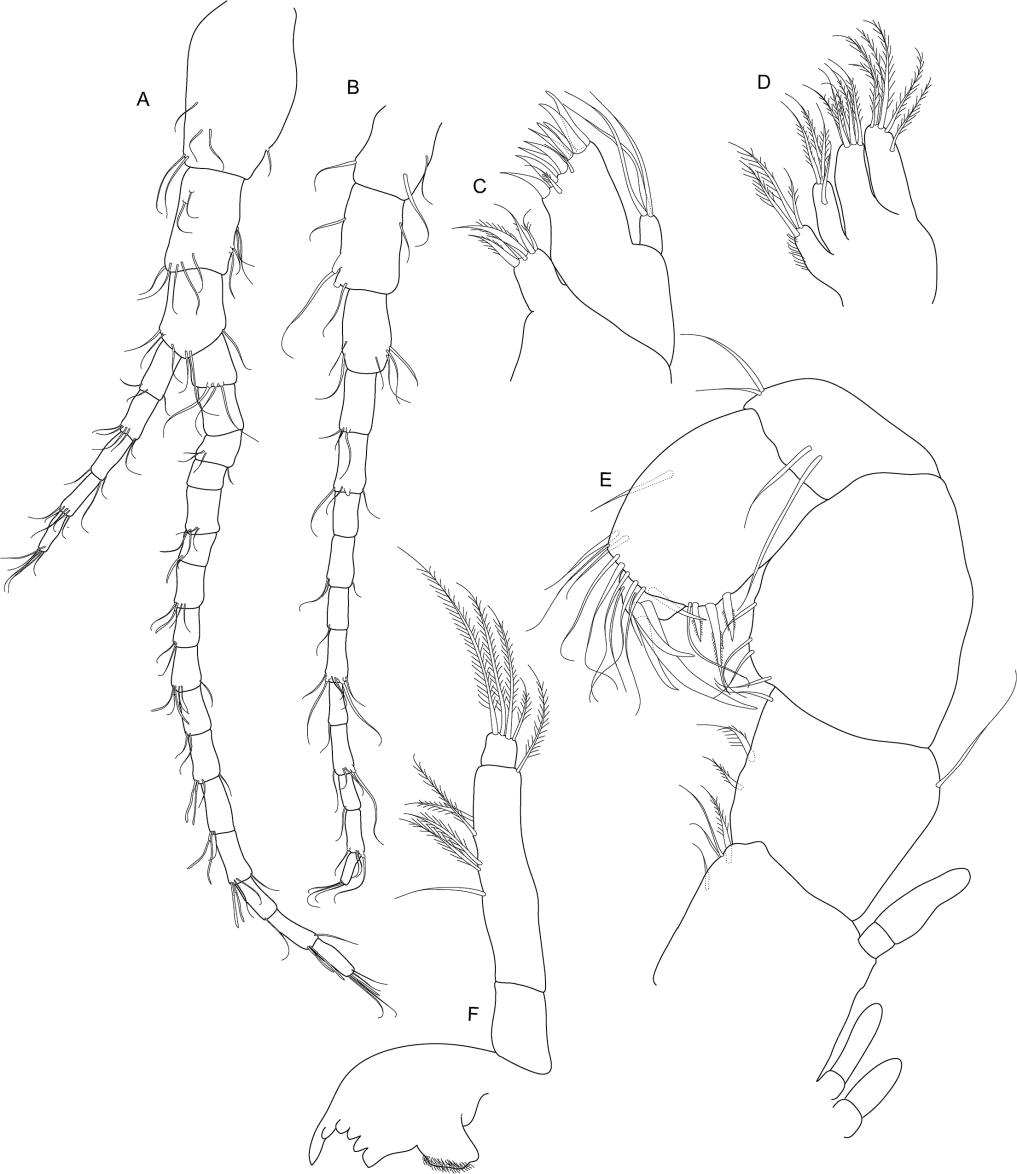
*Koonunga tatiaraensis*, sp. nov., holotype male, 3.67 mm, SAMA C C8422: A, antennule; B, antenna; C, maxilla 1; D, maxilla 2; E, maxilliped; F, mandible.

**Remarks:** *K*. *hornei* sp. nov. is most similar to *K*. *tatiaraensis* sp. nov., in that individuals are small (generally <10 mm), with no defined eyes, sparse body pigment concentrated around the head and posterior pleonites, possess short antennae (~1/2 body length) and in males the sternal process lacks a posterior projection. Adult *K*. *hornei* sp. nov. individuals were generally larger than *K*. *tatiaraensis* sp. nov. (~7 mm vs ~4 mm), and the male individual described for *K*. *tatiaraensis* sp. nov., which showed adult morphology for pleopods 1–2, did not possess a sensory scale on the antennule (Fig 12A and 12B). As well as some setal differences on the last pleonite, the habitats of the two species are seemingly different with *K*. *hornei* sp. nov. collected widely from caves, sinkholes and bores between Penola and Mount Gambier, South Australia and *K*. *tatiaraensis* sp. nov. restricted to groundwater systems between Keith and Padthaway, South Australia (Fig 1).

Water chemistry measured in groundwater observation wells (GAM 078, KLE 011, "middle bore", MTM 060 (obswell numbers) presented as avg±SE): Temperature 15.8–0.11°C, Specific Conductance- 1174.5–91.56 mS/cm, Salinity 0.61–0.049 ppt, pH 7.5–0.08, Dissolved Oxygen 28.4–6.64% saturation (for raw data see S1 Table).

**Distribution:** South-east South Australia (Fig 1), found in caves, sinkholes and groundwater observation bores.

**Etymology:** Named after Peter Horne in honour of his extensive efforts in diving and specimen collection in caves and sinkholes in the Mount Gambier area, which has contributed a substantial number of *Koonunga* specimens to the collections of the South Australian Museum.

***Koonunga tatiaraensis* Leijs & King, sp. nov**.

(Figs 12–15)

**Fig 13:**
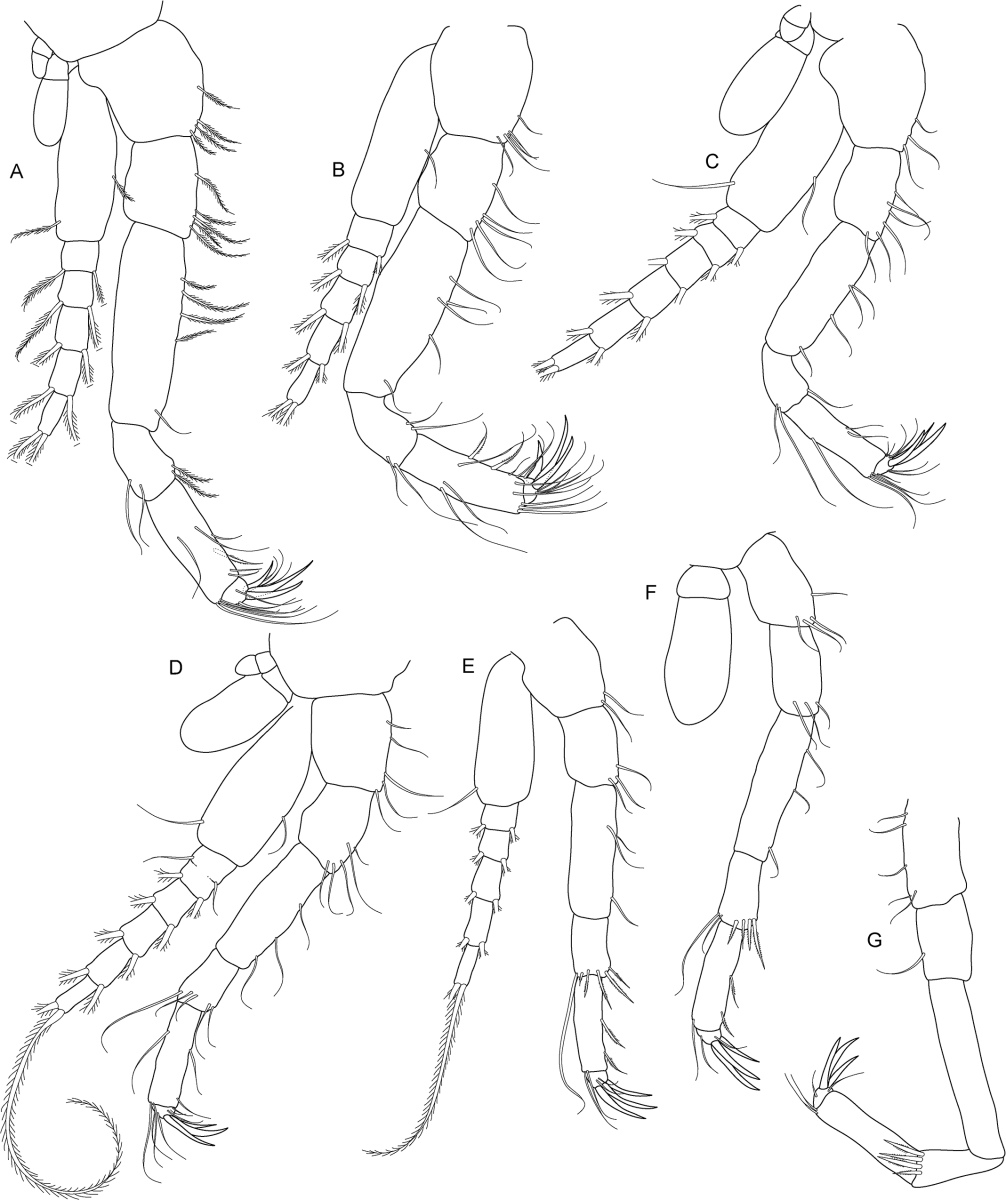
*Koonunga tatiaraensis*, sp. nov., holotype male, 3.67 mm, SAMA C8422: A, pereopod 1; B, pereopod 2; C, pereopod 3; D, pereopod 4; E, pereopod 5; F, pereopod 6; G, pereopod 7.

**Fig 14:**
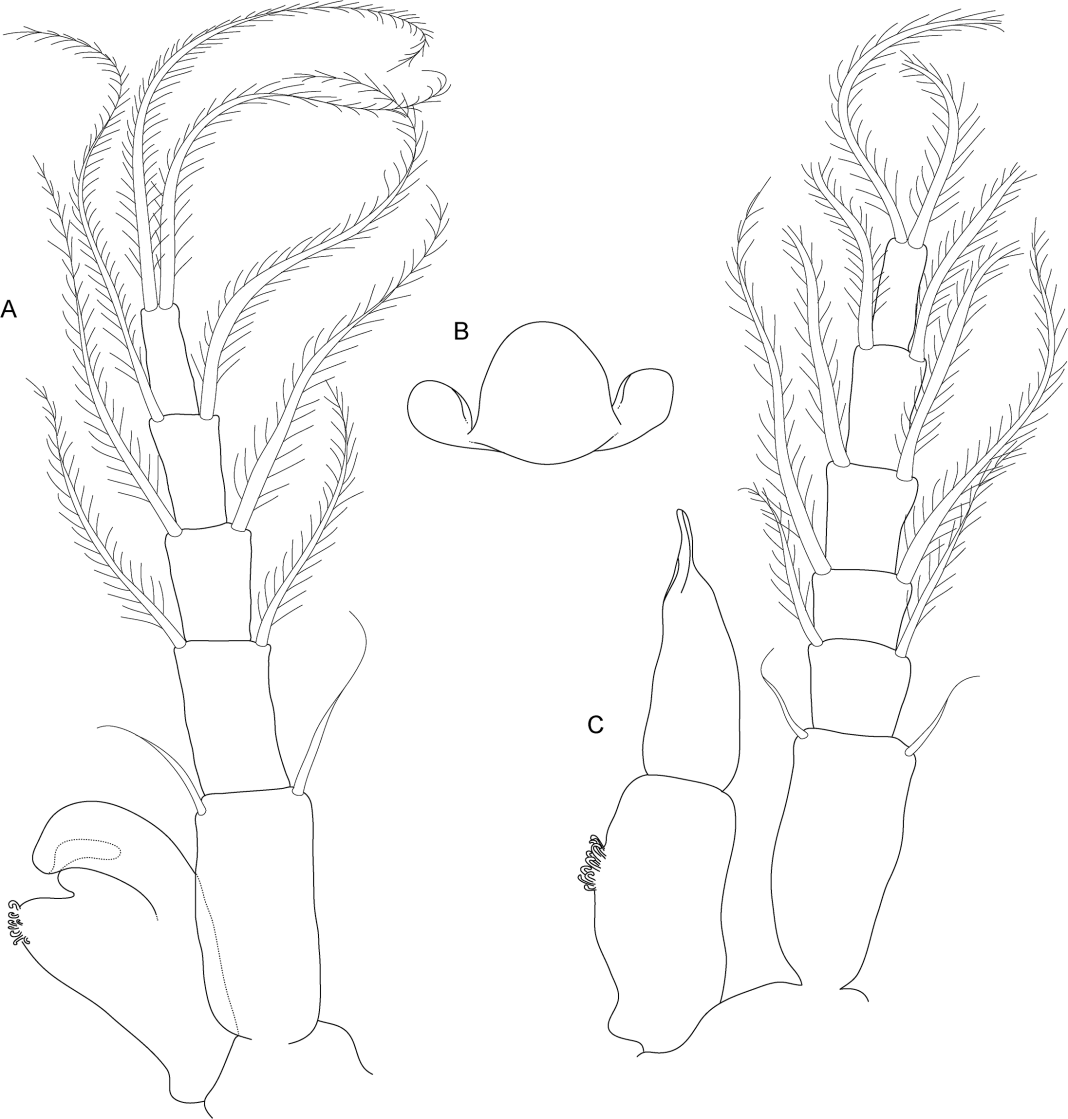
*Koonunga tatiaraensis*, sp. nov., holotype male, 3.67 mm, SAMA C C8422: A, pleopod 1; B, endopod (petasma) ventral view; C, pleopod 2.

**Fig 15:**
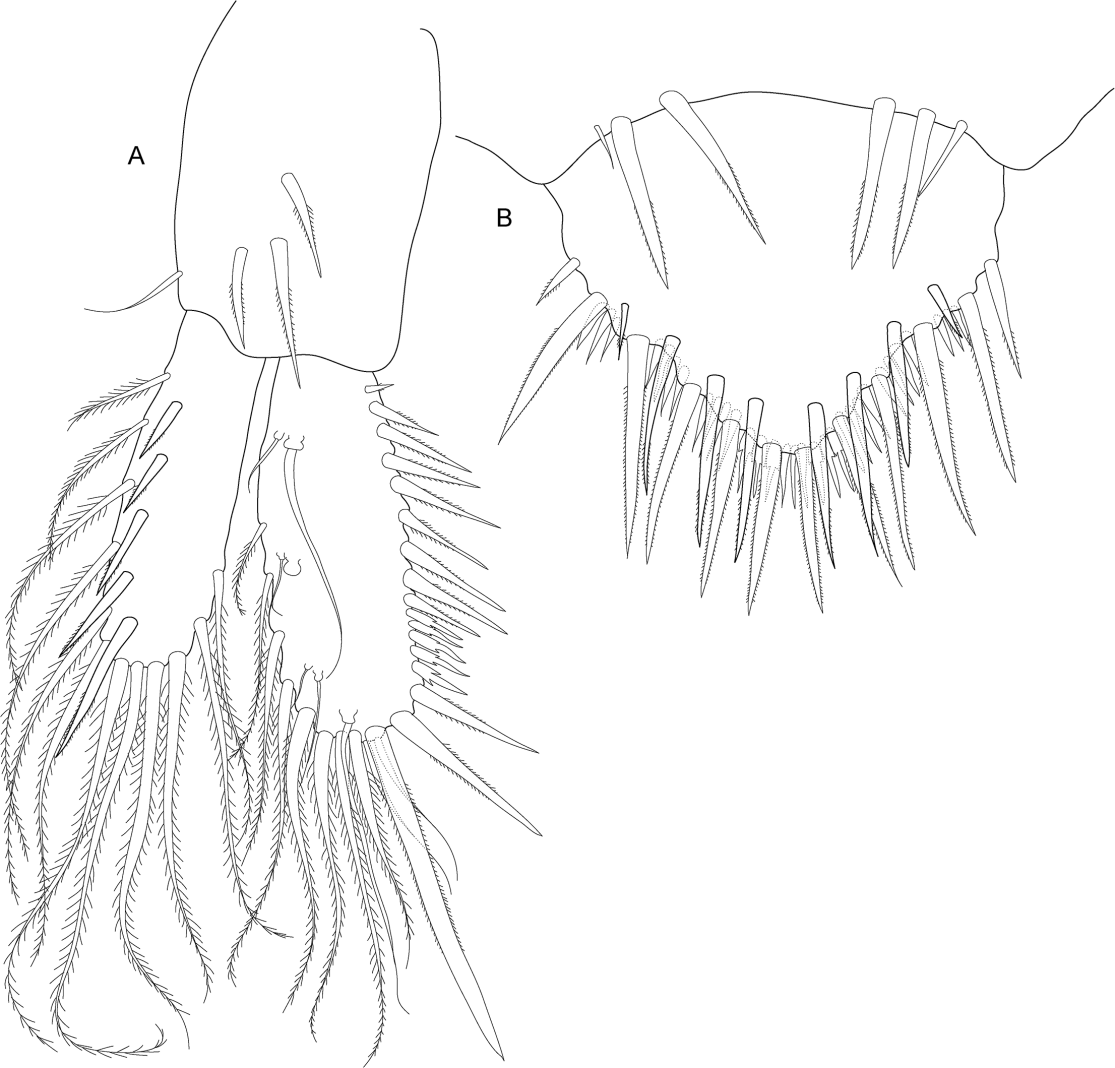
*Koonunga tatiaraensis*, sp. nov., holotype male, 3.67 mm, SAMA C C8422: A, uropod; B, telson.

LSID urn:lsid:zoobank.org:act:09208FC5-EBFA-4822-886C-728D0CA84647

**Material examined:** *Holotype*. SAMA C8422, 1 male, WRG110 near Keith, SA, observation bore, 04-May-2012, 36.29325°S, 140.595°E, coll. R. Leijs. *Paratypes*. SAMA C8420, SAMA C8421, 3 females, collected with holotype.

**Additional material examined:** see Table 1.

**Molecular diagnosis:** Molecular diagnostic characters: ten fixed amino acid differences relative to *K*. *crenarum*, five are unique for the species. Amino acid positions are relative to the first amino acid downstream of the M414 primer site S at positions 15, 161; N at position 30; V at positions 94, 120, 152, 169 (Table 3).

**Description:** Holotype male (SAMA C8422)

Length 3.67 mm. Antennule length 1.94 mm. Antenna length 1.41 mm. Head length 0.57 mm. Pereon length 1.56 mm. Pleon length 1.32 mm. Telson length 0.21 mm. Pleon (excluding telson) 0.37mm.

Head rectangular, with short pointed rostrum. Defined eyes absent, pigment present and diffuse across the anterior margin of the head near antero-lateral.

Antennule (Fig 12A) as long as 0.53 times body length; peduncle with 3 articles, basal article as long as following two combined, articles 1 and 2 outer margins with small plumose setae, simple setae and type 5 setae, inner margins with 5–7 setae, of which distal setae most robust; outer flagellum with 16 articles, about 3.5 times the length of inner flagellum, sensory organ not present on article 2, with aesthetascs present on every second article; inner flagellum with 5 articles

Antenna (Fig 12B) length slightly more than 0.7 of antennule length; peduncle with 4 articles (basal article not dissected), basal article very short, articles 2–4 elongate and rectangular shaped, article 3 longest, articles distally with 2–3 type 5 setae; flagellum with 11 articles, type 5 setae on every second article.

Mandible (Fig 12F) palp with 3 articles; article 2 greatly enlarged, about 2 times length article 1, with 5 (LHS) and 3 (RHS) plumose setae along inner margin; article 3 small, rounded, with 4 (LHS) and 6 (RHS) plumose setae apically. Base of mandible stout, ending in well-developed molar and incisor process. Molar process with small grinding surface surrounded by numerous spiniform setae. Incisor process of left mandible with six denticles, denticle nearest molar reduced. Incisor process of right mandible with five denticles. No evidence of secondary cutting plate or spine row.

First maxilla (Fig 12C) two lobed; outer lobe with small one segmented palp with three robust plumose setae, extremity of outer lobe obliquely truncated with 9 setulate robust setae, some stouter than others and 1–2 smaller setulate setae near outer (aboral) surface; inner lobe about ½ width of outer lobe with 1 robust seta near inner (oral) surface surrounded by 4 smaller setulate setae.

Second maxilla (Fig 12D) smaller than first maxilla, consisting of four lobes; inner lobe smallest with others increasing successively in length and width; inner lobe covered with fine simple setae, with 3 plumose setae distally; outer lobes with more numerous (4–6) slightly longer setae.

Maxilliped (Fig 12E) (thoracopod 1) stout, of 7 segments flexed posteriorly between articles 4 and 5; article 1 (coxa) shorter and wider than following segments with 2 epipodites near outer aboral corner; article 2 (basis) with exopodite of 2 joints resembling epipodites, exopodite not reaching past article 3, aboral margin with several simple setae near distal oral margin; article 3 (ischium) slightly expanded on outer distal corner, slightly wider and longer than article 2, with several long, fine plumose setae on inner margin and a single long plumose seta on outer aboral corner; article 4 (merus) inflated proximally, longer than any other segment, only slightly narrower than article 1; article 5 (carpus), small, with row of long setae along distal inner margin for inner half and on oral and aboral outer distal margin; article 6 (propodus) robust, slightly shorter than article 4, with 3 robust setulate setae on inner surface and row of long setae on aboral distal corner; article 7 (dactylus) small, rounded, with 4 strong claw-like robust setae (one large and three small) and long setae near inner margin.

Pereopods 1–7 (thoracopods 2–8) (Fig 13A–13G) similar in structure to maxilliped, progressively becoming more slender with all articles more elongate. Pereopods 1–6 slightly shorter than maxilliped, pereopods 5 and 6 shortest overall, pereopod 1 longest overall. Pereopods 1–6 coxa with two unequal epipodites as in maxilliped (not fully illustrated in Fig 13B, 13E and 13F). Pereopods 1–5 bases with multi-segmented exopodite consisting of large basal segment reaching well past ischium. Pereopod 6 basis without exopodite. Pereopod 7 without epipodite or exopodite. Pereopods 6–7 carpus distal margin with a row of strong plumose setae. Pereopods 1–7 dactylus with 3 claw-like robust setae, similar to maxilliped.

Pleopods 1–2 (Fig 14A–14C) with endopodites modified to form complex copulatory structure (petasma). Pleopod 1 endopodite of one article and joined by coupling hooks on lobe on inner margin (Fig 14A). Pleopod 2 endopodite of two articles of about equal length, each as long as endopodite of pleopod 1; basal article with coupling hooks on small pad at mid length (Fig 14C); Distal segment apically pointed, hollowed out on distal, inner margin to form a concave depression directed towards body; Sternal process anterior median lobe dome shaped; Sternal process posterior median lobe absent; Sternal process lateral lobes shorter than median lobe; PL3-5 of similar structure, lacking endopodites but with multi-segmented exopodites consisting of basal segment and flagellum of 4-6segments, each bearing 2 long plumose setae.

Uropod with peduncle stout (Fig 15A), rectangular, about 1.4 times as long as wide, and slightly shorter than rami, with 3 robust setulate setae and 1 smaller seta on dorsal surface and near outer margin; outer ramus slightly longer than inner ramus, with around 12 long plumose setae along inner and outer margins and row of 5 short strong upturned setulate setae near outer dorsal margin; inner ramus with around 8 long plumose setae along outer and proximal margin; inner proximal margin with 3 dorsally directed terminal robust setae, the two smaller setae ½ length of terminal seta; dorsal inner margin with row of 8 strong upturned curved setulate setae for about proximal 2/3 length steadily increasing in size followed by a row of 5 smaller tridentate setae ending at first terminal seta; dorsal outer margin on inner ramus with row of often paired type 5 setae.

Telson (Fig 15B) triangular, with narrow rounded apex and slightly convex lateral margins, length (excluding spines) slightly less than width, margins with three arrangements of spines (in order dorsal to ventral): 8 posterodorsally directed robust setulate setae, 12 posteroventrally directed setulate robust setae, ~24 posteriorly directed short setae about 1/3 in length of middle posteroventrally directed setae and with two pairs of 3 tridentate setae subapically.

**Remarks:** See morphology remarks for *K*. *hornei* sp. nov. None of the *K*. *tatiaraensis* sp. nov. males examined possessed an antennular organ. Zeidler [16] described the development of the antennular organ in *K*. *crenarum* as size dependent so it is possible that we have not examined large enough males, however Zeidler [16] noted that in *K*. *crenarum* males with developed pleopodal petasmata also possessed an obvious antennular organ and all male *K*. *tatiaraensis* sp. nov. individuals examined here had fully developed pleopodal petasma.

Water chemistry measured in groundwater observation wells (PAR 043, MAR 029, GLE 103, GLE 100, MAR 002 (obswell numbers) presented as avg ± SE): Temperature 17.6–0.15°C, Specific Conductance- 3060.8–336.60 mS/cm, Salinity 1.6–0.18ppt, pH 7.0–0.07, Dissolved Oxygen 45.9–5.87% saturation (for raw data see S1 Table).

**Distribution:** South-east South Australia (Fig 1), found only in groundwater observation bores between Keith and Padthaway.

**Etymology:** Named after the district of the type locality.

## Discussion

This paper recognised three new species of *Koonunga* from a relatively large and more or less continuous aquifer in the south-east of South Australia. The species are diagnosed using molecular analyses and morphology. Interestingly, the species distributions partly overlap, they vary with respect to the amount of body pigmentation, and presence or absence of eyes. The well supported phylogeny of the species, combined with molecular dating analyses and the morphological variation (in body pigmentation and presence or absence of eyes), and potentially restricted dispersal capabilities of the stygobitc species *K*. *hornei* and *K*. *tatiaraensis* in particular, enables correlation with palaeo-geographic and climatic data to infer plausible scenario(s) for the evolution of these species in the area.

Strikingly, our molecular clock analyses suggest that species divergence times, (Table 4, nodes 1–3) estimated at 10.7–22.3 Mya (5.38–33.80: 95% interval), and their distributions do not correlate with the age of the landscape, which is estimated to have been above sea level less than 1.1 Mya [19]. This is a conundrum because the Anaspidacea (which includes *Koonunga*) are freshwater organisms. Multiple scenarios might offer an explanation to this problem.

First, our molecular clock calibration may not be appropriate for *Koonunga*. For the time calibration of our molecular phylogeny we implemented an uncorrelated log-linear relaxed clock rate prior of 0.0105 substitutions per site per million years [32] which was based on a large number of independent rate estimates. We also calibrated our tree using a prior of 1.1 Mya on the root node to explore whether average rates in the phylogeny would be within an expected range for this crustacean genus. The outcome of a mean rate of 0.062 substitutions per site per million years is far beyond any of the values used for calculation of the invertebrate clock rate in Papadopoulou et al [32]. There are also no plausible reasons to suspect such an increased molecular rate. We therefore suggest that time calibration in our analyses would not be an issue.

Second, the simplest explanation is that an ancestral species inhabited the marine environment, and that speciation occurred there. These species then all independently and more or less passively colonised the freshwater as a result of a marine regression that progressively occurred in the area since about 1.1 Mya [19]. This scenario reflects the two-step model of stygofauna evolution in connection with marine regressions [39, 40, 41], whereby marine benthic species colonise marine interstitials and then subsequently get stranded and adapt to freshwater as consequence of marine regressions. The problems with this model for *Koonunga* are twofold. Currently all species within the Anaspidacea are found in freshwater environments and there are no good reasons implying extinctions as recent as < 1Mya of *Koonunga* from the marine environment. Additionally, with a progressive marine regression and subsequent stranding of species one would expect that this would be reflected in the phylogeny: those species that stranded first (*K*. *tatiaraensis*) would take the position as a sister lineage to all other species. The species phylogeny (Fig 2), however, shows a pattern that is opposite to what is expected under this model, namely the coastal species, e.g. *K*. *crenarum*, in a basal position and *K*. *tatiaraensis* in a proximal position. Therefore, an evolutionary scenario based on marine ancestral species is not plausible.

Third, we propose a scenario that assumes the species evolved from a freshwater epigean-adapted *Koonunga* ancestor that inhabited creeks, rivers and lacustrine habitats. The major speciation period for *Koonunga* estimated here at 10.7–22.3 Mya (Table 4) coincides with a period of dramatic regional landscape changes. Concisely, it involved the marine Murray Basin in which thick deposits of limestone were laid down (Murray Basin/Mount Gambier Limestone, > 10 Mya [19]); these limestone deposits form the aquifer matrix in which three of the *Koonunga* species occur. Subsequently a marine regression occurred, with the ancestral Murray River flowing through the Lake Hindmarsh area and draining out to sea through the Glenelg River. Uplift cut off the drainage of the Murray River and formed Lake Bungunnia, 1.2–2.4 Mya [42, 19], which was an extensive freshwater habitat that would have been suitable for *Koonunga*. Breeching of the tectonic dam east of the Mount Lofty Ranges formed the present flow path of the Murray River and combined with considerable drying of the climate, circa 0.9 Mya, caused the end of Lake Bungunnia. Currently, apart from the River Murray, there is no permanent fresh surface water in the wider area that would support *Koonunga*.

The Western Highlands, situated to the south-east of the Murray Basin (Fig 1) has been a much more palaeographically stable environment in which permanent flowing creeks and rivers draining inland, east and south would have provided suitable habitat for *Koonunga* for a long period. The current distribution of the *Koonunga* species is south and west of the Western Highlands. For a freshwater epigean origin of *Koonunga* we suppose that diversification happened in the Western Highlands area and that isolation within drainages could have led to the formation of one or more endemic species, similar to paramelitid amphipod species in the Pilbara region of Australia [43]. Prior to significant drying of the climate which started around 1 Mya [44], the inland lakes and rivers (possibly including the Murray River) could have supported epigean species of *Koonunga*. During the aridification of inland Australia that had extremes during Pleistocene glacial maxima [44], inland epigean species may have become extinct as surface waters became more ephemeral, unless they were able to colonise and find refugia in subterranean environments. Suitable subterranean environments were probably provided by the underlying Murray Basin/Mount Gambier Limestones which were likely to have contained a more or less continuous freshwater karst aquifer. Numerous ‘run-away’ holes, which are depressions in the landscape or even creeks that disappear in the limestone, are still present in the Tatiara district near Keith and Bordertown and these may have assisted passive colonisation of the aquifer by ancestors of *K*. *tatiaraensis* during heavy rainfall and major aquifer recharge events. Further south, south east of Penola (Fig 1) the flat landscape, which includes numerous lagoons associated with high groundwater tables, which still exist today, could have forced ancestors of *K*. *hornei* and *K*. *crenarum* underground during severe dry spells.

The effects of aridificaction in the south of the Western Highlands almost certainly were not as severe as more inland areas and permanent water would have ensured survival of epigean lineages such as *K*.*cursor* and *K*. *allambiensis*.

In summary, in this third scenario we propose that ancestral lineages of the *Koonunga* species, distributed in catchments of the Western Highlands were all epigean and that due to severe aridification in the last 1 My some of the species independently made the transition to the subterranean environment and subsequently lost their pigment and eyes.

Regressive evolution of pigment and eyes is a process that may happen in the course of a few generations [45, 46] and could be irreversible, as has been suggested for an eye pigment gene in stygobitic diving beetles [47]. With respect to regressive evolution, the species *K*. *hornei* and *K*. *crenarum* are interesting because they have overlapping distributions (Fig 1), but differ in size and in expression of stygobitic characters. *K*. *hornei* is a small stygobitic species without body pigment and eyes while *K*. *crenarum* is large, has some body pigment, but no eyes. The difference in size may be explained by ecological niche differentiation as has been shown for amphipods [48], whereby *K*. *crenarum* occupies the larger cavities in the Mount Gambier limestone karst aquifer and *K*. *hornei* also occupies the smaller fissures. The difference in the amount of body pigment, however, requires a different explanation. *K*. *crenarum* is most commonly found in the cenotes, large water filled collapsed caves which are exposed to daylight, and that were only formed after the Last Glacial Maximum (20ka) [14, 15]. It is plausible that *K*. *crenarum* and *K*. *hornei* colonised the aquifer at a similar time during the first major marine regression and climatic cooling/drying about 780 kya [49] and subsequently both species lost their eyes and body pigment. However, *K*. *crenarum* could have regained some of its body pigments, but significantly less than the epigean species *K*. *allambiensis* and *cursor* after the formation of the cenotes, as an adaptation to the more photic habitat, but not its eyes.

Alternatively, *K*. *crenarum* may never completely have lost its body pigmentation, perhaps due to an absence of chance destructive mutations in the genetic pathway leading to pigmentation. However, there are numerous observations of epigean crustacean species that partly inhabit the dark zone of springs or subterranean streams whereby the majority of the individuals in these habitats show an absence of body pigmentation, but they still have functional eyes, although there is tendency for eye reduction and eye colour to change from black to red and white (eg., *Asellus aquaticus* Say, 1818 [50], *Gammarus minus* (Linnaeus, 1758) [51] [45, 46]). Such species show a clear gradient in body pigmentation, eye colour and size, where these characters decrease with increased distance from the photic zone. This suggests that at least the phenotypic expression of loss of body pigmentation is a faster process than the complete loss of eyes.

For *Koonunga*, currently the genetics of body pigmentation and eyes is not known. Protas et al. [45] used *Asellus aquaticus*, a freshwater isopod that lives in caves as well in surface waters, as a model species to study the genetic basis of eye and pigment loss and found that eye loss is associated with a single gene, while eye reduction and body pigment involves multiple genes. If the genetic basis for these characters is similar in *Koonunga* then it is possible that mixing of unrelated lineages in *K*. *crenarum* could have led to the recovery, in part, of body pigmentation. Studying regressive evolution of pigment and eye genes in this group would be worthwhile to further elucidate the mechanism of eye and pigment evolution and to test the validity of the above presented evolutionary scenarios.

To conclude, molecular phylogenetic analyses combined with palaeogeographic and climatic reconstructions have shown that ancestral *Koonunga* species were most likely living in epigean habitats and that some of these lineages passively made transitions to subterranean habitats driven by climatic drying and cooling less than 1 Mya. The cenotes that formed after the Last Glacial Maximum resulted in a dramatic change in photic conditions from a completely dark cave environment to that of an epigean freshwater lake environment, in which *K*. *crenarum*, as an adaptation to the presence of light, may have regained some of its body pigmentation. Cenotes are therefore potentially ideal environments to study the possibility of bi-directionality of regressive evolution in cave adaptation.

## Supporting Information

S1 Table Water Chemistry of groundwater wells. (XLSX)

## References

[1] Calman WT. On the classification of the Crustacea Malacostraca. The Annals and Magazine of Natural History 1904; 7: 144–158.

[2] Brooks HK. On the fossil Anaspidacea, with a revision of the classification of the Syncarida. Crustaceana 1962; 4: 229–242.

[3] Jarman SN, Elliott NG. DNA evidence for morphological and cryptic Cenozoic speciations in the Anaspididae, `living fossils' from the Triassic. Journal of Evolutionary Biology 2002; 13: 624–633.

[4] Chilton C. Notes on a fossil shrimp from Hawkesbury sandstones. Journal of the Royal Society New South Wales 1929; 62: 366–368.

[5] Thomson GM. On a freshwater schizopod from Tasmania. Transactions of the Linnean Society London (Zoology) 1894; 6: 285–303.

[6] Schram FR. Fossil Syncarida. Transactions of the San Diego Society of Natural History 1984; 20: 189–246.

[7] Thomson GM. Notes on Tasmanian Crustacea, with descriptions of new species. Proceedings of the Royal Society Tasmania 1893; 1: 45 76.

[8] Sayce OA. Description of a remarkable new crustacean with primitive malacostracan characters. Victorian Naturalist 1907; xxiv. No. 7, pp. 117–120, 1907. Reprinted (1908) Annals and Magazine of Natural History, 8: 350–355.

[9] Schminke HK. *Psammaspides williamsi* gen. n. sp. n. ein Vertreter einer neuen Familie mesopsammaler Anaspidacea (Crustacea, Syncarida) 1974; Zoologica Scripta 3: 177–183.

[10] Poore GCB. Crustacea: Malacostraca: Syncarida, Peracarida: Isopoda, Tanaidacea, Mictacea, Thermosbaenacea, Spelaeogriphacea In, Houston, W.W.K. & Beesley P.L. (eds) Zoological Catalogue of Australia 2002; Vol 19.2A Melbourne: CSIRO Publishing, Australia xii 434pp.

[11] Noodt W. Estudios sobre crustaceos de aguas subterraneas III, Crustacea Syncarida de Chile central 1963; Investigaciones Zoológicas Chilenas 10: 151–167.

[12] Camacho AI, Valdecasas AG. Global diversity of syncarids (Syncarida; Crustacea) in freshwater. Hydrobiologia 2008; 595:257–266.

[13] Nicholls GE. *Micraspides calmani*, a new syncaridan from the west coast of Tasmania 1931; Journal of the Linnean Society London (Zoology) 37: 473–488.

[14] Drummond FH. The syncarid Crustacea, a living link with remote geological ages. Australian Museum Magazine 1959; 13: 63–64.

[15] De Dekker P. New records of *Koonunga cursor* Sayce, 1908 (Syncarida, Anaspidacea). Transactions of the Royal Society of South Australia 1980; 104: 21–25.

[16] Zeidler W. A new species of crustacean (Syncarida: Anaspidacea: Koonungidae) from sinkholes and caves in the south-east of South Australia. Transactions of the Royal Society of South Australia 1985; 109: 63–75.

[17] Williams WD. Freshwater Crustacea. Pp.63-111. *In* Williams, W.D. (ed.) “Biography and Ecology in Tasmania”, Monographiae Biologicae. 1974; 25.

[18] Mustafa S, Lawson JS. Review of Tertiary Gambier Limestone Aquifer Properties, Lower South-east. Department of Water, Land and Biodiversity Conservation Report South Australia. 2002; 86 pp, Adelaide.

[19] McLaren S, Wallace MW, Gallagher SJ, Miranda JA, Holdgate GR, Gow LJ, et al Palaeogeographic, climatic and tectonic change in southeastern Australia: the Late Neogene evolution of the Murray Basin. Quaternary Science Reviews 2011; 30: 1086–1111.

[20] Imbrie J, Hays JD, Martinson DG, McIntyre A, Mix AC, Morley JJ, et al The orbital theory of Pleistocene climate: support from a revised chronology of the marine δ18O record *In*: Berger A.L., et al (Ed.), Milankovitch and Climate, Part I. D. Reidel, Dordrecht, 1984; pp. 269–305.

[21] Murray‐Wallace CV, Brooke BP, Cann JH, Belperio AP, Bourman, RP. Whole‐rock aminostratigraphy of the Coorong coastal plain, South Australia: towards a 1 million year record of sea‐level highstands. Journal of the Geological Society, London 2001; 158, 111‐124.

[22] Australian Stratigraphic Units Database. Available: http://dbforms.ga.gov.au/pls/www/geodx.strat_units. Accessed 12 May 2014.

[23] Webb JA, Grimes KG, Lewis ID. Volcanogenic origin of cenotes near Mt Gambier, southeastern Australia. Geomorphology 2010; 119: 23–35.

[24] Peltier WR, Fairbanks RG. Global glacial ice volume and Last Glacial Maximum duration from an extended Barbados sea level record. Quaternary Science Reviews 2006; 25: 3322–3337.

[25] Bou C, Rouch R. Un nouveau champ de recherche sur la faune aquatique souteraine. Comptes-Rendus de l’Académie des Sciences de Paris 1967; 265: 369–370.

[26] Chomczynski P, Mackey K, Drews R, Wilfinger W. DNAzol: a reagent for the rapid isolation of genomic DNA. Biotechniques, 1997; 22: 550–3. 9067036 10.2144/97223pf01

[27] Folmer O, Black M, Hoeh W, Lutz R, Vrijenhoek R. DNA primers for the amplification of mitochondrial cytochrome *c* oxidase subunit I from metazoan invertebrates. Molecular Marine Biology and Biotechnology 1994; 3, 294–299. 7881515

[28] Tamura K, Dudley J, Nei M, Kumar S. MEGA4: Molecular Evolutionary Genetics Analysis (MEGA) software version 4.0. Molecular Biology and Evolution 2007; 24: 1596–1599. 17488738 10.1093/molbev/msm092

[29] Swofford DL. ‘PAUP*: Phylogenetic Analysis Using Parsimony (* and other methods). Version 4.0b8. 2001. (Sinauer: Sunderland, MA.).

[30] Ronquist F, Teslenko M, van der Mark P, Ayres DL, Darling A, Höhna S, et al MrBayes 3.2: efficient Bayesian phylogenetic inference and model choice across a large model space. Systematic Biology 2012; 61: 539–42. 10.1093/sysbio/sys029 22357727 PMC3329765

[31] Drummond AJ, Rambaut A. BEAST: Bayesian evolutionary analysis by sampling trees. BMC Evolutionary Biology 2007; 7, 214 10.1186/1471-2148-7-214 17996036 PMC2247476

[32] Papadopoulou A, Anastasiou I, Vogler AP. Revisiting the Insect Mitochondrial Molecular Clock: The Mid-Aegean Trench Calibration. Molecular Biology and Evolution 2010; 27: 1659–1672. 10.1093/molbev/msq051 20167609

[33] Brower A. Rapid morphological radiation and convergence among races of the butterfly *Heliconius erato* inferred from patterns of mitochondrial DNA evolution. Proceedings of the National Academy of Sciences USA 1994; 91: 6491–6495. 10.1073/pnas.91.14.6491 PMC44228 8022810

[34] Rodríguez F, Oliver JF, Marín A, Medina JR. The general stochastic model of nucleotide substitutions. Journal of Theoretical Biology 1990; 142: 485–501. 2338834 10.1016/s0022-5193(05)80104-3

[35] Yang Z. Among-site rate variation and its impact on phylogenetic analyses. Trends in Ecology and Evolution, 1996; 11: 367–372. 21237881 10.1016/0169-5347(96)10041-0

[36] Rambaut A, Drummond A J. Tracer v1.4. 2007; Available: http://beast.bio.ed.ac.uk/Tracer. [verified November 2013].

[37] Garm A. Revising the definition of the crustacean seta and setal classification systems based on examinations of the mouthparts setae of seven species of decapods. Zoological Journal of the Linnean Society 2004; 142: 233–252.

[38] Packard AS. The Syncarida, a group of Carboniferous Crustacea. American Naturalist 1885; 19: 700–703.

[39] Notenboom J. Marine Regressions and the Evolution of Groundwater Dwelling Amphipods. Journal of Biogeography 1991; 18: 437–454.

[40] Boutin C., Coineau N.. 1990 Regression Model, Modèle biphase d’évolution et origine des micro-organismes stygobies interstitiels continentaux. Revue de Micropaléontologie 33 (3/4): 303–322.

[41] Coineau N., Boutin C.. 1992 Biological processes in space and time. Colonization, evolution and speciation in interstitial stygobionts In Camacho A.I. ed., The natural History of Biospeleology. Museo Nacional de Ciencias Naturales, CSIC Ed., Madrid, Monografias 7: 423–451.

[42] Stephenson AE. Lake Bungunnia–a Plio-Pleistocene megalake in southern Australia. Palaeogeography, Palaeoclimatology, Palaeoecology 1986; 57, 137–156.

[43] Finston TL, Johnson MS, Humphreys WF, Eberhard SM, Halse SE. Cryptic speciation in two widespread subterranean amphipod genera reflects historical drainage patterns in an ancient landscape. Molecular Ecology 2007; 16, 355–365. 17217350 10.1111/j.1365-294X.2006.03123.x

[44] Byrne M, Yeates DK, Joseph L, Kearney M, Bowler J, Williams MAJ, et al Birth of a biome: insights into the assembly and maintenance of the Australian arid zone biota. Molecular Ecology 2008; 17, 4398–4417. 10.1111/j.1365-294X.2008.03899.x 18761619

[45] Protas ME, Trontelj P, Nipam H Patel NH.Genetic basis of eye and pigment loss in the cave crustacean, *Asellus aquaticus* . Proceedings of the National Academy of Sciences 2011; 108: 5702–5707. 10.1073/pnas.1013850108 PMC3078413 21422298

[46] Carlini DB, Manning J, Sullivan PG, Fong DW. Molecular genetic variation and population structure in morphologically differentiated cave and surface populations of the freshwater amphipod *Gammarus minus* . Molecular Ecology 2009; 18, 1932–1945 10.1111/j.1365-294X.2009.04161.x 19434810

[47] Leys R, Cooper SJB, Strecker U, Wilkens H. Regressive evolution of an eye pigment gene in independently evolved eyeless subterranean diving beetles. Biology Letters 2005; 1: 496–499. 10.1098/rsbl.2005.0358 PMC1626372 17148242

[48] Fišer C, Blejec A, Trontelj P. Niche-based mechanisms operating within extreme habitats: a case study of subterranean amphipod communities. Biology Letters 2012; 8: 578–581, 10.1098/rsbl.2012.0125 PMC3391468 22513281

[49] Sandiford M, Quigley M, De Broekert P, Kakica S. Tectonic framework for the Cenozoic cratonic basins of Australia. Australian Journal of Earth Sciences 2009; 56: S5–S18.

[50] Linnaeus C. Systema Naturae, Ed 10, vol I 1758; 824 pp. Salvii, Holmiae.

[51] Say T. An account of the crustacea of the United States Journal of the Academy of Natural Sciences of Philadelphia 1818; 1: 374–457.

